# Long-lasting improvements in episodic memory among subjects with mild cognitive impairment who received transcranial direct current stimulation combined with cognitive treatment and telerehabilitation: a multicentre, randomized, active-controlled study

**DOI:** 10.3389/fnagi.2024.1414593

**Published:** 2024-06-18

**Authors:** Rosa Manenti, Francesca Baglio, Ilaria Pagnoni, Elena Gobbi, Elena Campana, Cristina Alaimo, Federica Rossetto, Sonia Di Tella, Chiara Pagliari, Andrea Geviti, Natale Salvatore Bonfiglio, Rocco Salvatore Calabrò, Vincenzo Cimino, Giuliano Binetti, Angelo Quartarone, Placido Bramanti, Stefano F. Cappa, Paolo Maria Rossini, Maria Cotelli

**Affiliations:** ^1^Neuropsychology Unit, IRCCS Istituto Centro San Giovanni di Dio Fatebenefratelli, Brescia, Italy; ^2^IRCCS Fondazione Don Carlo Gnocchi – ONLUS, Milan, Italy; ^3^Department of Psychology, Università Cattolica del Sacro Cuore, Milan, Italy; ^4^Service of Statistics, IRCCS Istituto Centro San Giovanni di Dio Fatebenefratelli, Brescia, Italy; ^5^IRCCS Centro Neurolesi “Bonino Pulejo”, Messina, Italy; ^6^MAC Memory Clinic and Molecular Markers Laboratory, IRCCS Istituto Centro San Giovanni di Dio Fatebenefratelli, Brescia, Italy; ^7^Università Degli Studi eCAMPUS, Novedrate, Italy; ^8^Istituto Universitario Studi Superiori IUSS, Pavia, Italy; ^9^IRCCS Fondazione Mondino, Pavia, Italy; ^10^Department Neuroscience and Neurorehabilitation, IRCCS San Raffaele Roma, Rome, Italy

**Keywords:** MCI, telerehabilitation, cognition, tDCS, transcranial direct current stimulation

## Abstract

**Background:**

In recent years, an increasing number of studies have examined the potential efficacy of cognitive training procedures in individuals with normal ageing and mild cognitive impairment (MCI).

**Objective:**

The aims of this study were to (i) evaluate the efficacy of the cognitive Virtual Reality Rehabilitation System (VRRS) combined with anodal transcranial direct current stimulation (tDCS) applied to the left dorsolateral prefrontal cortex compared to placebo tDCS stimulation combined with VRRS and (ii) to determine how to prolong the beneficial effects of the treatment. A total of 109 subjects with MCI were assigned to 1 of 5 study groups in a randomized controlled trial design: (a) face-to-face (FTF) VRRS during anodal tDCS followed by cognitive telerehabilitation (TR) (clinic-atDCS-VRRS+Tele@H-VRRS); (b) FTF VRRS during placebo tDCS followed by TR (clinic-ptDCS-VRRS+Tele@H-VRRS); (c) FTF VRRS followed by cognitive TR (clinic-VRRS+Tele@H-VRRS); (d) FTF VRRS followed by at-home unstructured cognitive stimulation (clinic-VRRS+@H-UCS); and (e) FTF cognitive treatment as usual (clinic-TAU).

**Results:**

An improvement in episodic memory was observed after the end of clinic-atDCS-VRRS (*p* < 0.001). We found no enhancement in episodic memory after clinic-ptDCS-VRRS or after clinic-TAU.

Moreover, the combined treatment led to prolonged beneficial effects (clinic-atDCS-VRRS+Tele@H-VRRS vs. clinic-ptDCS-VRRS+Tele@H-VRRS: *p* = 0.047; clinic-atDCS-VRRS+Tele@H-VRRS vs. clinic-VRRS+Tele@H-VRRS: *p* = 0.06).

**Discussion:**

The present study provides preliminary evidence supporting the use of individualized VRRS combined with anodal tDCS and cognitive telerehabilitation for cognitive rehabilitation.

**Clinical trial registration:**

https://clinicaltrials.gov/study/NCT03486704?term=NCT03486704&rank=1, NCT03486704.

## 1 Introduction

The Global Burden of Diseases study indicated that chronic neurological disorders (CNDs) are among the leading causes of disease burden worldwide, and the rehabilitation of CNDs appears to be a key factor for managing health problems, preventing disability, and reducing the social/economic impact of these diseases (GBD 2017 DALYs and HALE Collaborators, 2018; GBD 2019 Diseases and Injuries Collaborators, 2020; GBD 2021 Nervous System Disorders Collaborators, 2024).

Research suggests that early rehabilitation interventions for CNDs result in improved clinical outcomes; nevertheless, to date, rehabilitation is a very specialized and not always provided by the National Health Service, and it is typically performed in the advanced stage of the disease (Cieza et al., 2021).

Due to the growing need for early rehabilitation services for CNDs among the ageing population, a radical transformation of the health care system and the identification of new ways to strengthen care are necessary.

Cognitive deficits are a common consequence of neurodegenerative and other neurological disorders. The rehabilitation of neuropsychological deficits represents an expanding area of neurological rehabilitation (Taub et al., 2002; Stuss et al., 2008; Parsons, 2016; Maggio et al., 2023). Cognitive difficulties have gained increased amounts of attention in recent years. These disorders can cause significant personal, social, and functional burdens as well as difficulties with activities of daily living. Furthermore, non-pharmacological interventions to prevent and treat cognitive deficits in patients with neurodegenerative disease have been widely studied in recent years. Among non-pharmacological interventions, cognitive training is a potential approach for improving cognitive function and delaying cognitive decline (Woods and Britton, 1977; Cappa et al., 2003, 2005; Clare and Woods, 2004; Cotelli et al., 2006; Clare et al., 2010; Kortte and Rogalski, 2013; Bahar-Fuchs et al., 2013a,b, 2019; Gates and Sachdev, 2014; Hong et al., 2015; Clare, 2017; Rai et al., 2018; Kudlicka et al., 2023).

A critical aspect of cognitive rehabilitation programs is that the most promising interventions involve intensive in-person sessions that are unlikely to be cost-effective or feasible for large-scale implementation (Corbett et al., 2015; Matamala-Gomez et al., 2020; Realdon et al., 2023).

There is a need to provide alternative services dedicated to people at risk of developing neurocognitive disorders, services that can be responsive to the increased demand and at the same time reduce health care costs (Bharucha et al., 2009; Astell et al., 2019; Moyle, 2019). Equitable access to services, improved quality of care, continuous intervention, and promotion of self-management are some of the benefits that can result from the provision of digital medicine and telerehabilitation services (Cherney and van Vuuren, 2012; Cotelli et al., 2019; Maggio et al., 2024).

The delivery of rehabilitation via a variety of technologies appears to be an attractive approach for overcoming the limitations of high-intensity face-to-face (FTF) rehabilitation interventions (Brennan et al., 2011; Realdon et al., 2016; Pitt et al., 2019; Isernia et al., 2020; Rossetto et al., 2023; Pagliari et al., 2024). Moreover, telerehabilitation has been shown to have comparable outcomes to traditional in-person service delivery (Brennan et al., 2002; Rosen, 2004; Poon et al., 2005; Mashima and Doarn, 2008; Kairy et al., 2009; Cherney and van Vuuren, 2012; Jelcic et al., 2014; Vermeij et al., 2016; Antonietti et al., 2017; Burton and O'Connell, 2018; Cotelli et al., 2019; Isernia et al., 2019; Alaimo et al., 2021).

Mild cognitive impairment (MCI) is a condition associated with memory loss and with risk of cognitive decline (Petersen et al., 1999; Petersen, 2004; Petersen et al., 2014; Livingston et al., 2017; Frisoni et al., 2023). In a previous randomized controlled trial (RCT) performed by our group (Manenti et al., 2020a), we reported that cognitive function rehabilitation intervention involving the FTF Virtual Reality Rehabilitation System (VRRS) led to improved memory, language, and visuo-constructive skills compared with FTF treatment as usual. In addition, in the same participants, a cognitive telerehabilitation intervention was associated with greater maintenance of the improvements achieved than home-based unstructured stimulation.

Neurorehabilitation is a rapidly renewing field, and the change is being fuelled by the introduction of cutting-edge technologies, such as digital health technologies and noninvasive brain stimulation techniques, which enable personalized treatment approaches.

In this regard, in recent years, the use of neuromodulation techniques, such as transcranial direct current stimulation (tDCS), has emerged because of their ability to modify cortical plasticity by increasing excitability in cortical neurons within a specific network, improving cognitive abilities (Brunoni et al., 2012; Dayan et al., 2013; Antal et al., 2017; Lefaucheur et al., 2017; Menardi et al., 2022) in neurodegenerative disease (Cotelli et al., 2012, 2020; Manenti et al., 2020b; Saxena and Pal, 2021). Studies have suggested that the use of noninvasive techniques coupled with cognitive intervention is more effective than cognitive training or noninvasive brain stimulation applied alone (Hsu et al., 2015; Brem et al., 2020; Cotelli et al., 2020; Nissim et al., 2020; Pergher et al., 2022).

Consistent with this hypothesis, this study aimed to (i) evaluate the efficacy of Face-to-Face cognitive VRRS combined with anodal tDCS applied to the left dorsolateral prefrontal cortex (DLPFC) on episodic memory compared to placebo tDCS stimulation combined with Face-to-Face cognitive VRRS and FTF cognitive treatment as usual and (ii) determine how to prolong the beneficial effects of the treatment using a telerehabilitation approach. To achieve these objectives, we recruited a sample of subjects with MCI who underwent FTF VRRS combined with anodal or placebo tDCS followed by cognitive telerehabilitation, and we analysed the collected data along with those acquired in our previous study (Manenti et al., 2020a).

## 2 Materials and methods

**Figure 1:**
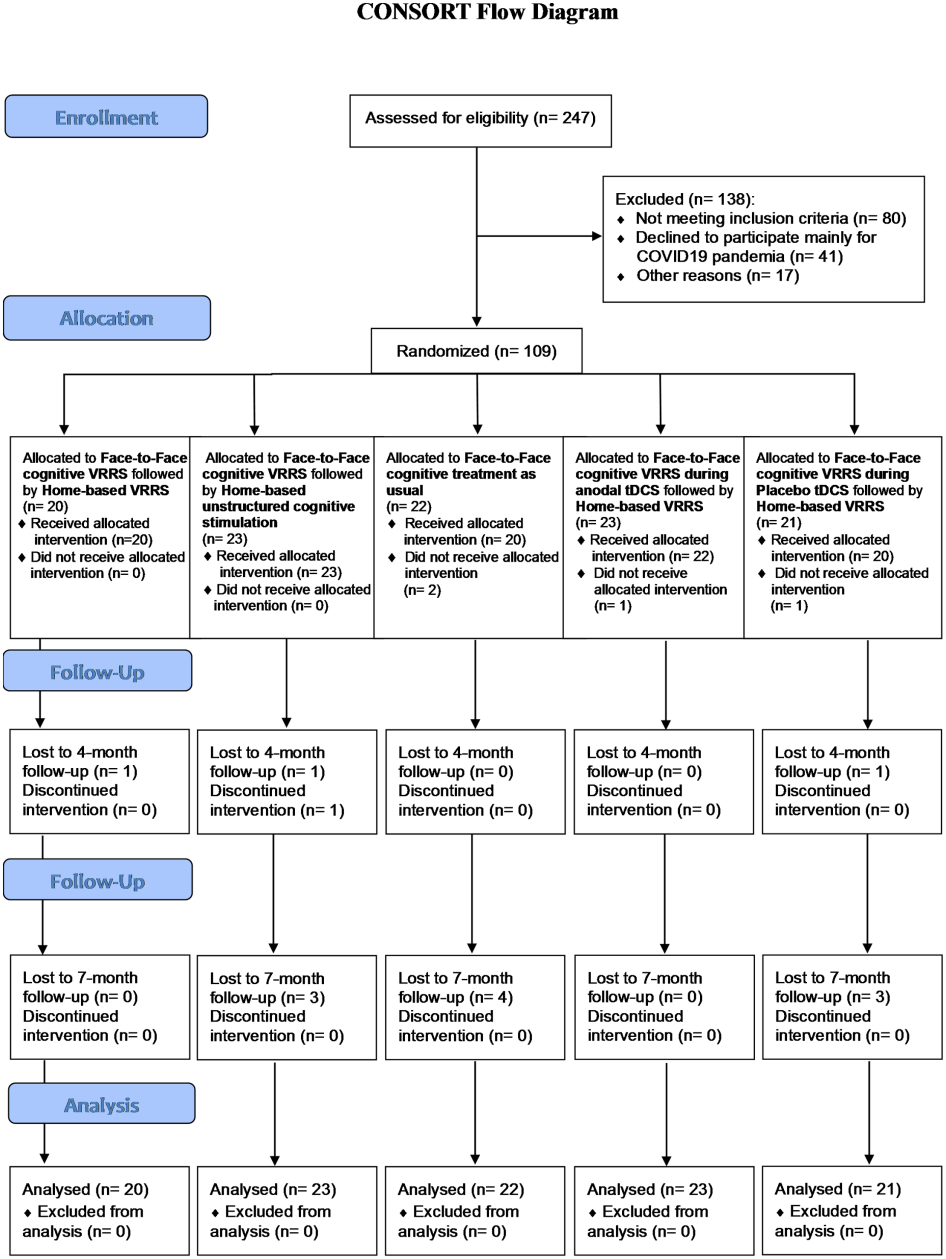
Flow chart showing study subject enrolment and sample processing.

Participants were recruited at the IRCCS Istituto Centro San Giovanni di Dio Fatebenefratelli of Brescia, the IRCCS Fondazione Don Carlo Gnocchi Onlus of Milan, and the IRCCS Centro Neurolesi Bonino-Pulejo of Messina from April 2018 to November 2022 (see Figure 1).

### 2.1 Study design

In this randomized, multicentre, active-controlled study, both the investigators and the outcome assessors were blinded to the treatment assigned to the participants. The study was approved by the local ethics committees (Ethics Statement numbers 48/2017 and 41/2020), conducted in accordance with the Declaration of Helsinki, and reported according to CONSORT guidelines (Boutron et al., 2008, 2017), and the trial was registered on clinicaltrials.gov (NCT number: NCT03486704). The CONSORT checklist is provided in the Supplementary materials.

All participants were fully aware of the aims of the study; written informed consent was obtained. A total of 109 subjects with MCI were randomly assigned to one of five experimental conditions: (a) Face-to-Face VRRS during anodal tDCS followed by cognitive telerehabilitation-TR (clinic-atDCS-VRRS+Tele@H-VRRS); (b) Face-to-Face VRRS during placebo tDCS followed by cognitive telerehabilitation (clinic-ptDCS-VRRS+Tele@H-VRRS); (c) Face-to-Face VRRS followed by telerehabilitation (clinic-VRRS+Tele@H-VRRS); (d) Face-to-Face VRRS followed by at-home unstructured cognitive stimulation (clinic-VRRS+@H-UCS); and (e) Face-to-Face cognitive treatment as usual (clinic-TAU).

Stratified randomization was performed by AG and NSB based on age and scores on the Mini Mental State Examination (MMSE; Folstein et al., 1975). Details of the allocated group were given to the researcher who wrote the treatment on cards contained in sequentially numbered, opaque, and sealed envelopes.

**Figure 2:**
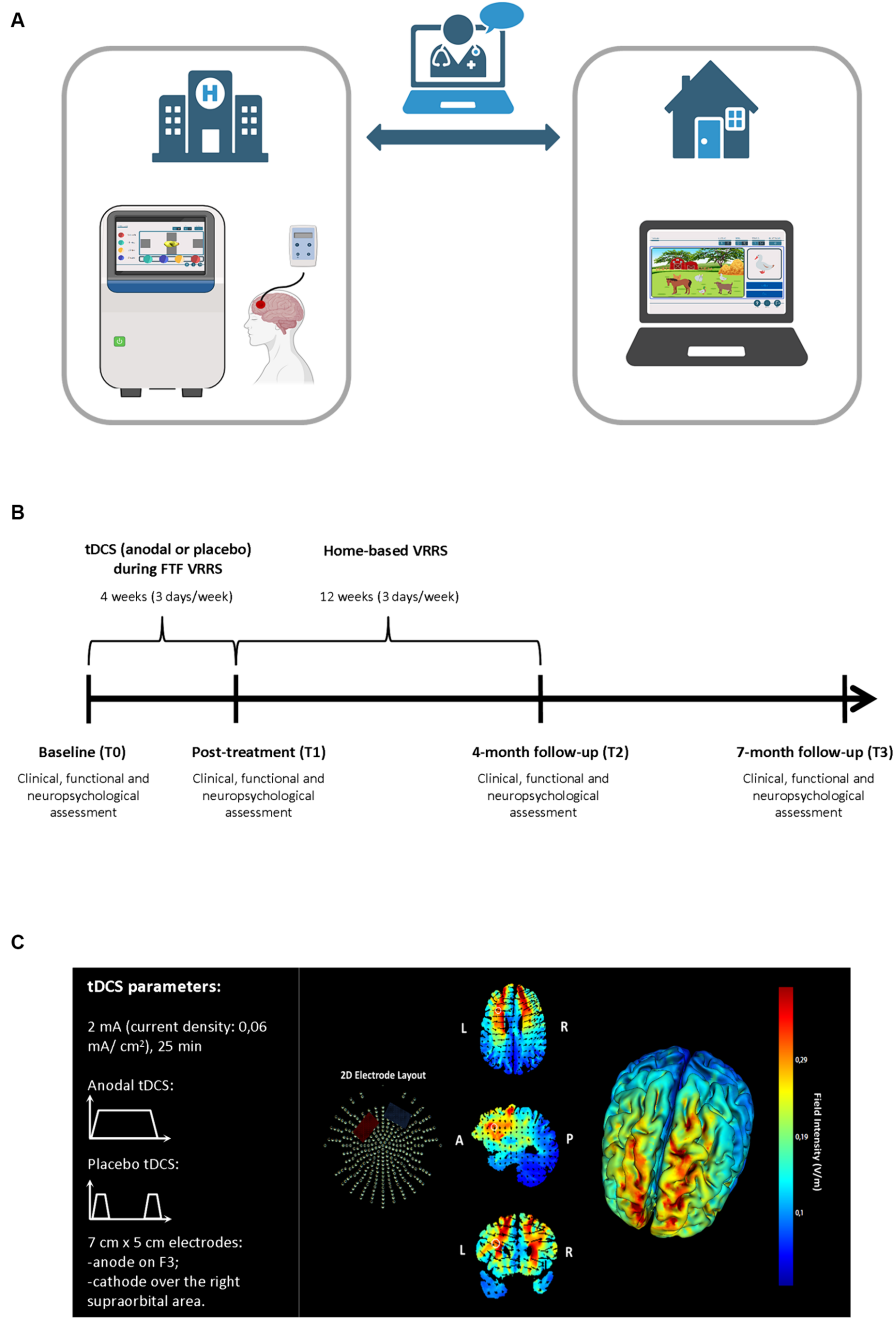
Experimental procedure for the face-to-face cognitive virtual reality rehabilitation system combined with anodal tDCS followed by cognitive telerehabilitation (clinic-atDCS-VRRS+Tele@H-VRRS) and for face-to-face cognitive virtual reality rehabilitation system combined with placebo tDCS followed by cognitive telerehabilitation (clinic-ptDCS-VRRS+Tele@H-VRRS). **(A)** Face-to-face (FTF) cognitive virtual reality rehabilitation system (VRRS) combined with tDCS followed by cognitive telerehabilitation. **(B)** Timeline for the experimental protocol of the FTF cognitive virtual reality rehabilitation system (VRRS) combined with anodal or placebo tDCS followed by cognitive telerehabilitation. **(C)** Current flow model of tDCS montage (anode over F3 and cathode over the right supraorbital area), using two 7×5 cm sponge pads represented in axial, sagittal and coronal views from the Male 1 model in the Soterix HD Targets software (Soterix Medical). Arrows represent the direction of current flow.

The original study protocol (Ethic Statement number 48/2017; Manenti et al., 2020a) was amended (Ethics Statement number 41/2020) by adding two experimental groups (clinic-atDCS-VRRS+Tele@H-VRRS; clinic-ptDCS-VRRS+Tele@H-VRRS) to evaluate the efficacy of cognitive VRRS combined with anodal tDCS applied to the left DLPFC compared to that of placebo tDCS stimulation combined with VRRS on episodic memory (Marion and Althouse, 2023). See Figure 2 Panel A.

The study protocol was carried out with no changes from the above amendments.

### 2.2 Participants

A total of 109 older adults who met the Petersen criteria for MCI (Petersen, 2011) and who were followed up annually for at least 2 years before enrolment were recruited. The inclusion criteria were as follows: (a) subjective complaints by the subject, a reliable informant or an expert clinician; (b) defective performance in at least one cognitive domain; (c) MMSE score greater than or equal to 24/30 (Folstein et al., 1975); (d) global Clinical Dementia Rating (CDR) score less than or equal to 1 (Morris, 1997); (e) preservation of functional autonomy; (f) absence of criteria for a diagnosis of dementia according to the Diagnostic and Statistical Manual of Mental Disorders (DSM-5; American Psychiatric Association, 2014); and (g) absence of depressive and anxiety symptoms. The exclusion criteria were as follows: other neurological or psychiatric disorders, visual or auditory perception disorders, history of traumatic brain injury, brain tumor or stroke, and alcohol abuse. No other cognitive training was administered during the duration of the present study (from baseline to the last follow-up assessment). Any contraindication for tDCS, such as a history of seizures, major head trauma, past brain surgery, brain metal implant, or a pacemaker, excluded the participant from the allocation to groups involving tDCS application.

The sample size calculation was based on a prior study assessing the effect of VRRS on MMSE scores in patients with Alzheimer’s disease (AD; Jelcic et al., 2014). Considering a significance level (α) of 0.05, a power (1-β) = 80 (two-tailed independent t test), and a dropout rate of 35%, the estimated sample size was twenty participants for each group.

### 2.3 Assessment procedures

Detailed records of previous medical events/visits and current medication, the Clinical Dementia Rating (CDR) scale (Morris, 1997), the Edinburgh Handedness Inventory (EHI; Oldfield, 1971), and the Cognitive Reserve Index questionnaire (CRIq; Nucci et al., 2012) were completed exclusively at the baseline assessment. Moreover, a comprehensive clinical, functional, and neuropsychological evaluation (approximately 90 min) was carried out for all groups at baseline (T0), at the end of FTF treatment (T1, 1 month from baseline), and at four (T2) and 7 months (T3) from baseline by expert neuropsychologists blinded to the treatment allocation of the participants.

Clinical and functional assessments included the Everyday Memory Questionnaire (EMQ; Sunderland et al., 1986; Calabria et al., 2011) for the evaluation of subjective memory complaints, basic (BADL) and instrumental activity of daily living (IADL) scales (Katz, 1983; Lawton and Brody, 1988) to assess the degree of autonomy in activities of daily living, the Geriatric Depression Scale (GDS; Yesavage et al., 1982) for depressive symptoms, the Neuropsychiatric Inventory (NPI; Cummings et al., 1994; Binetti et al., 1998) for neuropsychiatric symptoms and the Quality of Life in Alzheimer’s Disease (QOL-AD) scale (Bianchetti et al., 2017) for a measure of quality of life.

The standardized neuropsychological battery comprised the Mini Mental State Examination (MMSE; Folstein et al., 1975) for the assessment of global cognition as well as the following cognitive tests, which covered a broad range of cognitive abilities: the Rey Auditory Verbal Learning Test (RAVLT) for immediate and delayed recall (Carlesimo et al., 1996); the Free and Cued Selective Reminding Test (FCSRT; Frasson et al., 2011) and the Rey–Osterrieth complex figure (ROCF) test-recall (Caffarra et al., 2002) for episodic memory; Raven’s Colored Progressive Matrices for nonverbal reasoning (Basso et al., 1987); verbal fluency (phonemic and semantic; Novelli et al., 1986) and action and object naming subtests from the Battery for Aphasic Deficit Analysis (BADA; Miceli et al., 1994) for language production; the ROCF test-copy (Caffarra et al., 2002) for visuo-constructive abilities; and the Trail Making Test (TMT) part A and part B (Giovagnoli et al., 1996) for attention and executive functions (Lezak et al., 2012; see Tables 1, 2 for details).

**Table 1:** Sample characteristics.

	Total sample (*n* = 109)	Clinic-atDCS-VRRS+ Tele@H-VRRS (*n* = 23)	clinic-ptDCS-VRRS+ Tele@H-VRRS (*n* = 21)	Clinic-TAU (*n* = 22)	Clinic-VRRS + Tele@H-VRRS (*n* = 20)	Clinic-VRRS + @H-UCS (*n* = 23)	*p*-value^
Age, years	76.5 (4.2)	75.9 (4.6)	77.0 (4.0)	77.2 (4.0)	74.9 (3.4)	77.4 (4.6)	0.268
Education, years	10.1 (4.4)	9.8 (4.7)	9.3 (3.9)	10.0 (4.4)	11.4 (4.8)	9.8 (4.3)	0.687
Gender, males/females	52/57	10/13	14/7	8/14	14/6	6/17	0.013
Mini mental state examination – MMSE	27.0 (2.0)	26.9 (2.4)	27.2 (2.1)	26.9 (1.7)	26.9 (2.0)	27.1 (2.0)	0.961
Edinburgh handedness inventory (EHI)	78.9 (32.0)	63.8 (49.0)	76.8 (36.4)	88.8 (14.1)	79.9 (20.5)	85.5 (21.9)	0.311
Clinical dementia rating scale (CDR)	0.5 (0.1)	0.5 (0.1)	0.6 (0.2)	0.5 (0.1)	0.6 (0.2)	0.5 (0.1)	0.639
Cognitive reserve index – questionnaire (CRI-q)							
CRI-total score	111.0 (18.9)	112.6 (17.9)	104.6 (16.1)	109.6 (18.5)	115.1 (21.1)	113.2 (20.7)	0.422
CRI-education	103.6 (20.4)	101.9 (19.4)	104.2 (13.6)	105.9 (14.8)	99.5 (34.6)	106.1 (14.6)	0.969
CRI-working activity	103.3 (22.6)	106.4 (20.4)	102.8 (16.8)	101.2 (22.2)	102.6 (29.0)	103.1 (24.7)	0.959
CRI-leisure time	113.5 (22.7)	119.9 (19.3)	103.5 (23.1)	114.6 (21.6)	108.5 (25.2)	119.6 (21.7)	0.068

^ANOVA test for normally distributed variables; Kruskal-Wallis test for non-normally distributed variables; Chi-squared test for categorical variables. Mean are reported. Standard deviation between brackets.

**Table 2:** Descriptive statistics for clinical, functional, and neuropsychological evaluation.

	Clinic-atDCS-VRRS+ Tele@H-VRRS				Clinic-ptDCS-VRRS+ Tele@H-VRRS				Clinic-TAU				Clinic-VRRS+Tele@H-VRRS				Clinic-VRRS+@H-UCS				
	T0	T1	T2	T3	T0	T1	T2	T3	T0	T1	T2	T3	T0	T1	T2	T3	T0	T1	T2	T3	Cut-off
	Mean (SD)	Mean (SD)	Mean (SD)	Mean (SD)	Mean (SD)	Mean (SD)	Mean (SD)	Mean (SD)	Mean (SD)	Mean (SD)	Mean (SD)	Mean (SD)	Mean (SD)	Mean (SD)	Mean (SD)	Mean (SD)	Mean (SD)	Mean (SD)	Mean (SD)	Mean (SD)	
**Clinical and functional assessment**																					
EMQ	67.0 (28.1)	65.9 (27.3)	64.9 (28.4)	65.4 (29.4)	74.7 (30.4)	77.3 (29.2)	78.9 (32.1)	83.3 (30.9)	68.6 (25.4)	64.8 (25.7)	64.9 (25.4)	71.0 (23.0)	67.6 (22.0)	61.8 (23.3)	64.9 (26.7)	62.6 (22.9)	71.4 (25.5)	71.7 (27.4)	71.1 (25.1)	67.7 (23.0)	
QoL - AD																					
QoL- AD – Composite score	36.0 (3.2)	36.2 (3.4)	35.0 (3.8)	36.3 (4.0)	33.4 (4.0)	33.8 (4.6)	33.4 (4.1)	32.0 (4.4)	35.4 (4.2)	35.5 (3.3)	35.0 (4.2)	33.3 (4.6)	34.4 (3.7)	34.4 (4.0)	34.8 (3.9)	34.5 (4.9)	33.8 (4.9)	33.7 (5.1)	33.8 (6.0)	34.1 (6.3)	
QoL- AD – Patient score	36.7 (3.6)	36.8 (3.6)	35.6 (4.2)	36.8 (4.5)	33.8 (4.0)	34.2 (5.4)	33.8 (4.6)	32.2 (5.2)	35.9 (4.8)	36.3 (3.7)	35.4 (4.2)	34.0 (4.7)	35.3 (4.1)	34.9 (5.6)	35.9 (4.2)	35.7 (4.8)	33.5 (6.0)	33.8 (5.6)	34.0 (7.0)	34.3 (6.9)	
QoL- AD – Caregiver score	34.6 (6.9)	35.2 (5.7)	33.9 (5.2)	35.4 (5.2)	32.7 (5.8)	32.9 (4.6)	32.6 (5.1)	31.8 (4.9)	34.5 (4.7)	34.2 (4.5)	34.3 (5.0)	32.1 (5.3)	33.0 (6.4)	33.2 (5.8)	32.8 (6.6)	33.7 (5.9)	34.5 (4.7)	33.7 (5.0)	33.4 (5.6)	33.7 (7.1)	
NPI	5.1 (5.3)	5.6 (5.5)	5.0 (4.4)	5.0 (5.8)	7.1 (8.5)	7.2 (9.7)	7.5 (9.7)	10.6 (11.6)	4.0 (3.8)	3.9 (4.0)	4.1 (4.6)	5.1 (4.7)	6.4 (5.8)	6.0 (5.1)	6.7 (5.3)	5.6 (6.0)	5.2 (4.6)	5.7 (5.0)	6.8 (5.4)	7.8 (7.6)	
GDS	5.9 (3.6)	6.0 (3.5)	7.0 (4.9)	5.3 (4.1)	7.2 (4.0)	6.8 (3.3)	8.9 (4.2)	8.8 (4.6)	6.2 (4.6)	6.4 (4.4)	7.2 (4.6)	7.4 (3.9)	6.0 (4.4)	4.8 (4.1)	6.1 (5.3)	6.3 (4.7)	7.7 (5.2)	7.2 (5.0)	8.0 (5.7)	7.2 (6.1)	< 11
BADL	0 (0.2)	0 (0.2)	0.1 (0.5)	0.1 (0.4)	0.1 (0.3)	0.1 (0.3)	0.1 (0.3)	0.2 (0.4)	0 (0)	0 (0)	0 (0)	0 (0)	0.1 (0.3)	0 (0)	0.1 (0.2)	0.1 (0.2)	0 (0)	0.1 (0.3)	0 (0.2)	0.1 (0.3)	
IADL	0.2 (0.5)	0.3 (0.6)	0.5 (1.2)	0.5 (1.1)	0.3 (0.7)	0.5 (0.8)	0.7 (1.1)	0.9 (1.0)	0.2 (0.6)	0.2 (0.6)	0.2 (0.6)	0.4 (1.0)	0.6 (0.9)	0.3 (1.0)	0.4 (1.2)	0.5 (1.3)	0.3 (0.9)	0.3 (0.9)	0.4 (0.8)	0.4 (0.9)	
**Cognitive assessment**																					
Screening for dementia																					
MMSE	26.9 (2.4)	26.7 (2.4)	27.0 (2.5)	27.0 (2.5)	27.2 (2.1)	26.7 (1.9)	26.9 (1.9)	26.4 (2.5)	26.9 (1.7)	26.3 (2.4)	27.1 (1.9)	27.1 (2.3)	26.9 (2.0)	27.0 (2.4)	26.0 (3.2)	26.5 (2.3)	27.1 (2.0)	27.2 (2.2)	27.2 (2.1)	27.7 (1.7)	≥ 24
Non-verbal reasoning																					
Raven’s colored progressive matrices	27.6 (5.8)	28.1 (4.3)	27.3 (5.4)	27.0 (5.2)	27.0 (3.7)	28.9 (3.1)	28.3 (2.7)	28.1 (2.8)	26.5 (4.6)	27.4 (4.0)	26.9 (3.9)	27.0 (4.0)	28.9 (3.6)	29.0 (3.9)	29.3 (3.8)	29.4 (4.8)	25.6 (3.3)	26.3 (3.5)	26.5 (3.5)	25.9 (3.4)	> 17.5
Memory																					
RAVLT																					
RAVLT immediate recall	32.6 (14.1)	28.4 (10.7)	30.5 (13.7)	32.9 (14.2)	32.0 (13.6)	27.7 (10.8)	26.8 (11.6)	23.9 (13.4)	31.3 (7.9)	30.6 (6.6)	31.6 (7.5)	32.2 (8.8)	29.4 (6.6)	29.4 (8.4)	29.9 (9.4)	26.3 (7.6)	32.3 (7.7)	31.4 (7.0)	30.9 (9.0)	34.7 (8.7)	> 28.52
RAVLT delayed recall	5.4 (4.3)	4.7 (4.0)	5.0 (4.6)	5.2 (4.3)	5.5 (4.5)	4.8 (4.0)	4.4 (4.0)	3.7 (4.3)	4.0 (3.5)	4.2 (3.4)	4.3 (3.1)	4.8 (3.9)	4.1 (3.4)	4.5 (3.7)	4.2 (4.5)	3.9 (3.6)	4.8 (3.1)	4.3 (3.4)	4.6 (3.5)	5.6 (4.0)	> 4.68
FCSRT																					
FCSRT-IFR	20.5 (10.2)	23.0 (10.1)	21.7 (10.7)	23.2 (10.4)	20.4 (10.4)	21.0 (10.2)	21.4 (10.6)	18.4 (10.8)	19.0 (7.6)	19.8 (8.8)	20.2 (8.9)	21.1 (9.6)	18.6 (8.1)	20.4 (9.7)	18.3 (9.3)	18.6 (8.4)	20.0 (8.0)	23.8 (8.3)	22.7 (8.0)	22.9 (8.8)	> 19.59
FCSRT-ITR	32.9 (5.1)	33.3 (4.3)	32.9 (5.0)	33.2 (4.8)	32.4 (4.6)	33.1 (4.7)	32.8 (5.9)	31.8 (6.0)	33.2 (2.9)	32.5 (4.6)	33.0 (3.8)	33.1 (3.7)	32.0 (4.3)	31.8 (5.7)	31.1 (6.5)	32.2 (4.4)	32.2 (5.3)	33.2 (3.8)	33.3 (3.5)	33.9 (3.4)	≥ 35
FCSRT-DFR	7.1 (3.8)	7.7 (4.3)	7.8 (3.9)	7.9 (4.3)	6.2 (4.0)	6.7 (4.4)	6.7 (3.9)	5.9 (4.3)	5.9 (4.3)	6.8 (3.9)	6.3 (4.2)	6.6 (4.4)	5.8 (3.6)	6.6 (3.8)	5.7 (3.9)	6.8 (4.3)	6.6 (3.4)	7.9 (3.1)	8.2 (3.5)	8.2 (3.6)	> 6.31
FCSRT-DTR	11.0 (1.9)	10.9 (2.2)	10.7 (1.9)	10.9 (2.2)	10.5 (2.1)	10.9 (2.0)	10.5 (2.3)	10.2 (2.3)	10.7 (1.9)	10.9 (1.8)	10.7 (1.5)	11.1 (1.4)	10.3 (2.2)	10.2 (2.6)	10.2 (2.2)	10.4 (2.0)	10.4 (2.2)	11.0 (1.8)	11.0 (1.7)	11.3 (1.3)	≥ 11
FCSRT-ISC	0.9 (0.2)	0.9 (0.1)	0.9 (0.2)	0.9 (0.2)	0.8 (0.2)	0.9 (0.2)	0.9 (0.2)	0.8 (0.2)	0.9 (0.1)	0.8 (0.2)	0.8 (0.2)	0.9 (0.1)	0.8 (0.2)	0.8 (0.2)	0.7 (0.2)	0.8 (0.2)	0.8 (0.2)	0.8 (0.2)	0.8 (0.2)	0.9 (0.2)	≥ 0.9
ROCF - delayed recall	10.8 (5.9)	12.5 (6.8)	10.2 (5.8)	11.5 (5.8)	9.0 (5.7)	10.1 (7.3)	9.7 (6.5)	8.7 (5.7)	7.8 (5.5)	8.5 (5.5)	9.1 (6.7)	9.1 (7.5)	8.5 (6.9)	10.6 (8.3)	9.2 (7.1)	9.8 (6.9)	6.9 (4.8)	9.2 (5.3)	9.2 (4.1)	9.1 (5.4)	> 9.46
Language																					
Verbal Fluency, phonemic	31.8 (10.9)	32.1 (9.5)	31.7 (10.1)	32.4 (12.6)	28.3 (9.0)	29.9 (10.3)	30.7 (8.4)	28.8 (8.7)	29.5 (8.3)	32.5 (8.4)	31.6 (10.8)	31.5 (7.8)	29.6 (7.1)	32.0 (8.8)	29.6 (6.3)	31.5 (7.9)	29.2 (8.9)	33.0 (7.3)	30.6 (7.3)	31.1 (8.7)	> 16
Verbal Fluency, semantic	27.9 (9.0)	29.1 (9.1)	29.3 (9.1)	28.4 (10.1)	31.0 (12.4)	29.4 (11.1)	29.6 (13.2)	27.3 (12.0)	29.7 (7.3)	29.2 (6.3)	29.5 (7.4)	28.7 (6.2)	27.8 (5.7)	31.0 (6.8)	29.4 (6.0)	29.9 (7.0)	28.9 (6.5)	29.3 (7.7)	28.7 (6.3)	27.9 (5.0)	> 24
BADA – Objects naming	27.0 (2.9)	26.9 (2.7)	26.9 (2.6)	26.7 (2.9)	27.0 (2.9)	26.8 (3.2)	26.4 (3.7)	26.7 (3.5)	26.4 (2.9)	27.0 (2.0)	27.0 (2.1)	27.6 (2.2)	26.8 (2.3)	27.0 (2.5)	26.8 (2.4)	26.7 (2.9)	25.8 (2.9)	26.5 (2.9)	25.9 (3.2)	26.8 (2.0)	≥ 28
BADA – Actions naming	23.6 (3.0)	24.5 (2.8)	24.3 (2.7)	24.6 (2.3)	24.3 (3.6)	24.6 (3.3)	24.7 (2.8)	24.8 (3.2)	23.9 (3.4)	25.4 (2.3)	25.0 (2.3)	25.5 (2.3)	24.6 (2.9)	25.0 (2.4)	24.8 (2.5)	24.7 (2.7)	23.2 (3.3)	23.7 (3.4)	24.0 (3.4)	24.6 (3.3)	≥ 26
Attentional and executive functions																					
TMT, part A (s)*	48.8 (20.3)				51.9 (13.5)				52.5 (20.3)				57.4 (28.1)				60.3 (19.4)				< 94
TMT, part B (s)*	237.1 (157.5)				183.3 (99.5)				211.6 (117.9)				250.1 (143.7)				233.2 (133.8)				< 283
Visuo-constructional functions																					
ROCF - Copy	28.3 (5.6)	27.9 (6.3)	27.9 (6.1)	28.6 (4.9)	28.7 (5.4)	27.5 (5.9)	28.7 (6.4)	25.5 (6.0)	27.5 (5.9)	26.8 (6.1)	27.6 (6.2)	26.1 (8.1)	29.5 (6.1)	29.3 (5.8)	29.8 (5.1)	29.1 (6.5)	26.6 (5.0)	27.8 (4.7)	27.3 (5.1)	27.1 (4.8)	> 28.87

Raw scores mean are reported. Standard deviation between brackets. Cut-off scores according to Italian normative data are reported.S, seconds; T0, baseline; T1, Post-treatment; T2, 4 Months Follow-up; T3, 7 Months Follow-up; EMQ, Everyday Memory Questionnaire; QoL- AD, Quality of Life in Alzheimer’s Disease; NPI, Neuropsychiatric Inventory; GDS, Geriatric Depression Scale; BADL, Basic Activity of Daily Living scale; IADL, Instrumental Activity of Daily Living scale; MMSE, Mini Mental State Examination; RAVLT, Rey Auditory Verbal Learning Test; FCSRT, Free and Cued Selective Reminding Test; IFR, Immediate Free Recall; ITR, Immediate Total Recall; DFR, Delayed Free Recall; DTR, Delayed Total Recall; ISC=Index of sensitivity of cueing; ROCF, Rey–Osterrieth Complex Figure test; BADA, Battery for Analysis of Aphasic Deficits; TMT, Trail Making Test. *, administered only at Baseline (T0).

The participants who received FTF VRRS treatment (clinic-atDCS-VRRS+Tele@H-VRRS, clinic-ptDCS-VRRS+Tele@H-VRRS, clinic-VRRS+Tele@H-VRRS and clinic-VRRS+@H-UCS) underwent an assessment of system usability via the System Usability Scale (SUS; Brooke, 1996; Bangor et al., 2008, 2009; Peres et al., 2013) at T1. Moreover, we recorded the SUS scores at T2 in the subjects who underwent home-based treatment (clinic-atDCS-VRRS+Tele@H-VRRS, clinic-ptDCS-VRRS+Tele@H-VRRS, clinic-VRRS+Tele@H-VRRS and clinic-VRRS+@H-UCS).

### 2.4 Treatment

Participants received an FTF treatment that could be followed by a home-based treatment, according to their group allocation. The different types of FTF and home-based treatments outlined in this study protocol are described in the following paragraphs.

#### 2.4.1 Face-to-face treatment

All the participants enrolled in the study received 12 sessions of FTF cognitive training. According to the experimental group allocation, participants could undergo one of four treatments during FTF treatment: (a) FTF VRRS (clinic-VRRS) or (b) FTF cognitive treatment as usual (clinic-TAU); (c) FTF VRRS during anodal tDCS (clinic-atDCS-VRRS); (d) FTF VRRS during placebo tDCS (clinic-ptDCS-VRRS).

##### 2.4.1.1 Clinic-VRRS

Twelve 60-min sessions (over 4 weeks) of individualized cognitive rehabilitation using VRRS^1^ were administered to participants assigned to the clinic-VRRS.

The FTF cognitive VRRS included 12 exercises designed to enhance memory, visuospatial abilities, attention and executive functions. In each treatment session, the participant worked with six exercises, 10 min each, so that each exercise was completed six times over the 12 clinic-VRRS sessions. In all the sessions, except for the first and last sessions, strategies aimed at improving the subject’s performance were suggested by the researcher. Each training session ended with feedback on performance, and a detailed report was available. Clinic-VRRS treatment was tailored to the participant’s baseline characteristics: the starting level was adjusted using an adaptive staircase procedure. Progress was continuously monitored by the researcher, and each exercise adaptively progressed in difficulty.

##### 2.4.1.2 Clinic-TAU

Participants assigned to the clinic-TAU group received 12 60-min sessions of group cognitive stimulation in the clinic. During these group sessions, metacognitive training aimed at learning cognitive strategies and using external aids, reminiscence therapy, reality orientation therapy, and paper and pencil exercises was proposed by mental health professionals.

##### 2.4.1.3 Clinic-atDCS-VRRS or clinic-ptDCS-VRRS

To evaluate the efficacy of the cognitive VRRS combined with anodal tDCS applied to the left DLPFC, all of the individuals allocated to the clinic-atDCS-VRRS+Tele@H-VRRS or clinic-ptDCS-VRRS+Tele@H-VRRS group received tDCS stimulation over the left DLPFC (Anodal or Placebo, based on the assigned group) during the FTF VRRS cognitive training, starting at the beginning of the training (Figure 2 Panel A and B).

A tDCS stimulator (BrainStim, EMS, Bologna, Italy) delivered a constant low-intensity (2 mA) current for 25 min (with a ramping period of 10 s at the beginning and at the end of the tDCS session) through two saline-soaked sponge electrodes (7 cm x 5 cm, current density: 0.06 mA/cm^2^) (Bikson et al., 2016; Antal et al., 2017). An electroconductive gel was applied under the electrodes to reduce impedance, as in previous studies (Manenti et al., 2013; Sandrini et al., 2014, 2016). Participants and researchers were blinded to the tDCS condition applied: the anodal (active) or placebo stimulation mode was selected by entering a code.

The targeted region was the left DLPFC: the anode electrode was placed over F3, according to the 10–20 EEG international system, and the cathode electrode was located over the right supraorbital area. Figure 2 Panel C shows a graphical representation of the computerized modeling of tDCS-induced current flow.^2^ In the Placebo tDCS condition, the current was turned off 10 s after the beginning and was turned on for 10 s at the end of the stimulation period so that the participants could not distinguish between anodal and placebo stimulation (Manenti et al., 2013). Sensations induced by tDCS were assessed immediately after the stimulation session (Fertonani et al., 2015).

#### 2.4.2 Home-based treatment

According to the experimental group allocation, participants could undergo one of two treatments during home-based treatment: (a) cognitive telerehabilitation-TR (Tele@H-VRRS); (b) at-home unstructured cognitive stimulation (@H-UCS); or no home-based treatment.

##### 2.4.2.1 Tele@H-VRRS

After the end of FTF treatment, the participants assigned to Tele@H-VRRS received thirty-six 60-min sessions (3 sessions/week over 3 months) of home-based cognitive VRRS treatment (see text footnote 1, respectively). Twelve exercises different from those used in FTF VRRS training and designed to enhance memory, visuospatial abilities, attention and executive functions were selected. Each treatment session comprised six exercises (10 min each), and each exercise was completed 18 times over the thirty-six home-based VRRS sessions. Task difficulty adaptively progressed, and performance was continuously monitored by the researcher.

The VRRS has telerehabilitation functionalities, thus enabling the use of the same functionalities applied in the FTF treatment and the adjustment of exercise characteristics. Before beginning the home-based treatment, the researcher scheduled all 36 sessions of the individualized cognitive training exercise on the subject’s tablet. Moreover, a dedicated role-playing session was conducted with both the participant and his/her caregiver in order to familiarize with the technology. Specifically, the researcher shown they how to use the technological device and they were introduced to all the cognitive exercises included in the home-based treatment.

During home-based treatment, the researcher provided continuous assistance for technical difficulties, and the task difficulty of the individualized cognitive training exercises was remotely adapted once a week via a telerehabilitation platform by the clinician. Each participant received a home-based kit including a tablet that allowed access to a daily individualized training program, a detailed VRRS tablet manual, an exercise instructions booklet, and a diary.

##### 2.4.2.2 @H-UCS

Subjects assigned to @H-UCS were requested to work on detailed activities (paper and pencil exercises, creative manual activities, reading newspapers and magazines, watching documentaries, crosswords, and sudoku) 60 min a day, 3 times a week over 12 weeks (36 sessions in total). Participants received an instruction booklet and a diary.

### 2.5 Statistical methods

Summary statistics are expressed as the means and standard deviations. Comparisons of sociodemographic features, the scales completed exclusively at the baseline assessment and the system usability scale (SUS) scores between groups were evaluated by parametric (t tests) or corresponding nonparametric (Kruskal-Wallis) tests. The perceptions of sensation scores were compared between the anodal and placebo tDCS groups using the Mann–Whitney test.

The analyses were carried out using a modified intention-to-treat (mITT) approach. Specifically, given the lack of a unanimous consensus on this definition, as illustrated by Abraha and Montedori (2010), the inclusion criteria were based on the presence of at least a baseline assessment (four subjects had only a baseline assessment). According to the Cochrane guidelines for randomized trials,^3^ when dealing with a relatively low percentage of missing values [between 5% (small) and 20% (large); in our case, the percentage of missing values was 8% for all variables, except for TMT], it is reasonable to include participants with some missing values. Ultimately, the decision was made to retain the actual scores without replacement, as the type of analysis applied to the outcomes (generalized linear modeling, which allows the inclusion of all available observations without listwise deletion) and the reduced percentage of missing values in different conditions significantly reduce the risk of bias due to missingness in result interpretation.

Consistent with our first aim, we evaluated the efficacy of FTF cognitive VRRS combined with anodal tDCS compared to that of placebo tDCS stimulation combined with VRRS and of FTF cognitive treatment as usual for episodic memory (RAVLT and FCSRT scores).

For this purpose, we considered three groups of subjects—those who received clinic-atDCS-VRRS+Tele@H-VRRS, clinic-ptDCS-VRRS+Tele@H-VRRS or clinic-TAU—and compared their scores at all the time points (T0, T1, T2, T3). Based on the inherent distribution profiles of the variables (Gaussian, negative binomial, gamma, or Tweedie), we employed generalized linear mixed models (GLMMs) to examine the variations in scores between the three groups and across four distinct time points (T0, T1, T2, T3). Each model incorporated a distinct test score as the dependent variable, while independent variables included time, group, and the interaction term time*group. We treated time, group, and their interaction as fixed effects, while we considered the subjects as random effects. We implemented a repeated measure setting with an AR1 (first order autoregressive) covariance matrix. The AR1 structure explicitly models the correlation between repeated measures on the same subject, assuming that measurements taken closer in time are more highly correlated than those taken further apart. This structure helps in capturing the within-subject variability. Additionally, robust standard errors were requested for the fixed effects covariance estimates when necessary. This approach provides more reliable standard errors and test statistics when data assumptions like homoscedasticity or normality are violated. *Post hoc* assessments underwent correction using the sequential Bonferroni method. This method, differently from the classical Bonferroni method, is based on the ranking of *p*-values. Sequential Bonferroni-adjusted p-values were calculated. If this adjusted p-value was less than 0.05, it meant the result was still statistically significant even after the correction. When multiple comparisons were involved, we reported the sequential Bonferroni-adjusted p-value together with the non-adjusted one. Statistical analyses were performed using SPSS version 29.0, and R software (R Core Team, 2013) was used for the creation of graphical representations.

Consistent with our second aim, we assessed the possibility of prolonging the beneficial effects obtained after FTF treatment using a telerehabilitation approach. For this purpose, we considered all the experimental groups (clinic-atDCS-VRRS+Tele@H-VRRS, clinic-ptDCS-VRRS+Tele@H-VRRS, clinic-TAU, clinic-VRRS+Tele@H-VRRS, and clinic-VRRS+@H-UCS), and we compared the changes between T0 and T3 (i.e., the last follow-up visit). To achieve our objective, for the outcomes that improved significantly from T0 to T1, we calculated the difference (referred to as deltaT) between the scores recorded at time T3 and those at baseline (T0). DeltaT served as the dependent variable for our analysis. The distribution of deltaT closely resembled a beta distribution, characterized by its symmetrical shape and evidence of overdispersion. Consequently, we opted for beta regression, adjusting the distribution’s mean to zero to facilitate this process and considering group (with five different levels, one for each condition) as a predictor of the deltaT outcome. To enhance the interpretability of the regression coefficients, we applied an exponential transformation to them. The transformed coefficients should be understood as odds ratios, offering insights into the relationship between our independent variables and the observed changes in scores.

## 3 Results

A total of 247 subjects were evaluated for inclusion in this study. Ultimately, 138 subjects were excluded (80 subjects did not meet the inclusion criteria, 41 subjects declined to participate mostly due to the COVID-19 pandemic, and 17 for other reasons), whereas 109 subjects were deemed eligible for participation.

These 109 subjects were randomized into five experimental groups: 23 participants were allocated to the clinic-atDCS-VRRS+Tele@H-VRRS group; 21 participants were allocated to the clinic-ptDCS-VRRS+Tele@H-VRRS group; 20 participants were allocated to the clinic-VRRS+Tele@H-VRRS group; 23 subjects were allocated to the clinic-VRRS+@H-UCS group; and 22 participants were allocated to the clinic-TAU group (see Figure 1).

### 3.1 Participants

We enrolled 109 subjects with MCI, 72 (66%) with amnestic MCI and 37 (34%) subjects with nonamnestic MCI (Petersen, 2011). In particular, the current sample included (i) 36 subjects with amnestic single-domain MCI (aMCI-s), (ii) 36 subjects with amnestic multiple-domain MCI (aMCI-m), (iii) 26 subjects with nonamnestic single-domain MCI (naMCI-s), and (iv) 11 subjects with nonamnestic multiple-domain MCI (naMCI-m).

The five groups did not differ in terms of age (*p* = 0.268), education (*p* = 0.687), MMSE score (*p* = 0.961), EHI score (*p* = 0.311), CRI-Total Score (*p* = 0.422), CRI-Education score (*p* = 0.969), CRI-Working Activity score (*p* = 0.959), CRI-Leisure Time score (*p* = 0.068), or CDR scale score (*p* = 0.639), but there was a significant difference in sex (*p* = 0.013). See Table 1.

### 3.2 Face-to-face cognitive virtual reality rehabilitation system combined with anodal tDCS efficacy

Descriptive statistics for the clinical, functional, and neuropsychological evaluation scores at each time point are shown in Table 2.

**Figure 3:**
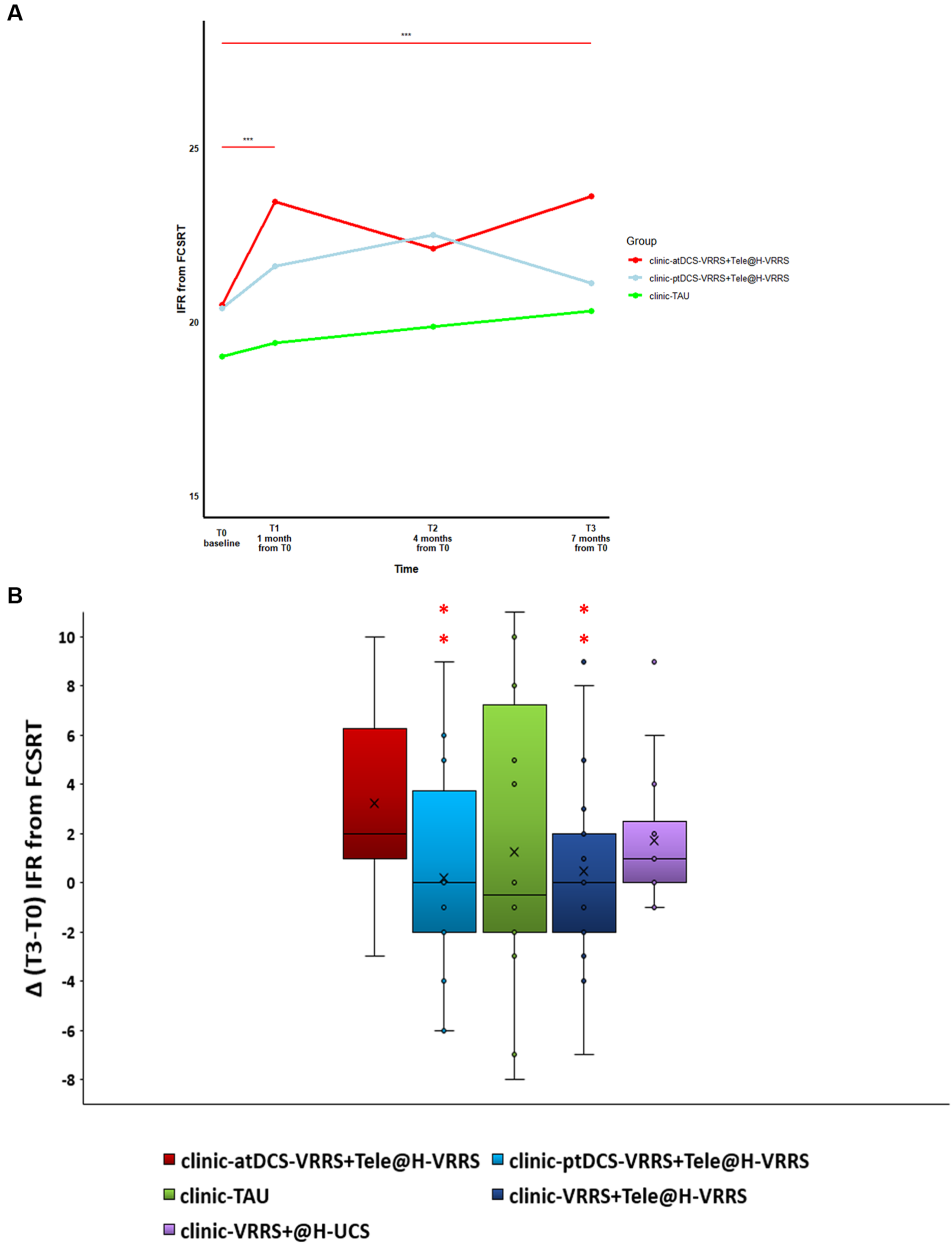
**(A)** Effects of face-to-face (FTF) cognitive virtual reality rehabilitation system (VRRS) combined with anodal tDCS followed by cognitive telerehabilitation (clinic-atDCS-VRRS+Tele@H-VRRS) vs. FTF cognitive VRRS combined with placebo tDCS followed by cognitive telerehabilitation (clinic-ptDCS-VRRS+Tele@H-VRRS) vs. FTF cognitive treatment as usual (clinic-TAU) on the immediate free recall (IFR) score of the Free and Cued Selective Reminding Test (FCSRT). Asterisks indicate significant comparisons for clinic-atDCS-VRRS+Tele@H-VRRS from T0 to T1 (*p* < 0.001) and from T0 to T3 (*p* < 0.001). **(B)** Long-term beneficial effects of face-to-face VRRS during anodal tDCS followed by cognitive telerehabilitation (clinic-atDCS-VRRS+Tele@H-VRRS). Box-plot of the delta deviations (differences between the scores recorded at time T3 and those at baseline T0) for the different conditions are reported. Asterisks indicate conditions that differed significantly from those of the clinic-atDCS-VRRS+Tele@H-VRRS group.

The results of the GLMMs are presented in Table 3. The only outcome that manifested significant differences between the three groups (clinic-atDCS-VRRS+Tele@H-VRRS, clinic-ptDCS-VRRS+Tele@H-VRRS, and clinic-TAU) from T0 to T1 and over the four time points (T0, T1, T2, and T3) was the immediate free recall (IFR) score of the FCSRT (interaction group*time: *p* = 0.002). In particular, the analyses revealed improvement from T0 to T1 (*p* = 1.03*10–5; p adj. = 0.00037) and maintenance at T3 (T0 to T3, *p* = 1.06*10–5; p adj. = 0.00037) only in the clinic-atDCS-VRRS+Tele@H-VRRS group (Figure 3A). Significant Group*Time interactions were also found for RAVLT-Immediate Recall (p = 0.002) and BADA-Objects Naming (*p* = 0.016), but none of the three groups improved or deteriorated significantly from T0 to T1. In particular, the analyses showed a decrease in RAVLT-Immediate Recall scores from T0 to T3 (*p* = 0.0005; p adj. = 0.017) only in the clinic-ptDCS-VRRS+Tele@H-VRRS group, while none of the groups showed a significant improvement or decrease over the four timepoints regarding BADA-Objects Naming.

**Table 3:** Generalized linear mixed models results for clinical, functional, and neuropsychological evaluation.

	Clinic-atDCS-VRRS + Tele@H-VRRS vs. clinic-ptDCS-VRRS + Tele@H-VRRS vs. clinic-TAU longitudinal evaluation at 4 time points		
	p_Time	p_Group	p_Time*Group
**Clinical and functional assessment**			
Everyday Memory Questionnaire (EMQ)	0.249	0.694	0.111
Quality of Life in Alzheimer’s Disease (QoL- AD) QoL- AD – Composite score	0.067	0**.025**	0.053
QoL- AD – Patient score	0.182	**0.018**	0.485
QoL- AD – Caregiver score	0.407	0.223	0.170
Neuropsychiatric Inventory (NPI)	0.120	0.148	0.451
Geriatric Depression Scale (GDS)	0.173	**<0.001**	0.057
Basic Activity of Daily Living scale (BADL)	1	0.730	0.830
Instrumental Activity of Daily Living scale (IADL)	0.051	0.486	0.709
**Cognitive assessment**			
Screening for dementia			
Mini Mental State Examination (MMSE)	0.143	0.905	0.778
Non-Verbal Reasoning			
Raven’s colored progressive matrices	0.087	0.449	0.440
Memory			
Rey Auditory Verbal Learning Test (RAVLT)			
RAVLT – Immediate recall	0.679	0.051	**0.002**
RAVLT – Delayed recall	0.575	0.789	0.802
Free and Cued Selective Reminding Test (FCSRT)			
FCSRT – Immediate free recall	**0.012**	0.569	**0.002**
FCSRT – Immediate total recall	0.965	0.950	0.386
FCSRT – Delayed free recall	**0.025**	0.384	0.888
FCSRT – Delayed total recall	0.650	0.868	0.437
FCSRT – Index of sensitivity of cueing	0.599	0.690	0.421
Rey–Osterrieth Complex Figure test (ROCF) – delayed recall	**0.014**	0.759	0.951
Language			
Verbal Fluency, phonemic	0.185	0.572	0.765
Verbal Fluency, semantic	0.075	0.855	0.281
Battery for analysis of aphasic deficits (BADA)			
BADA – objects naming	0.986	0.484	**0.016**
BADA – actions naming	**0.004**	0.787	0.680
Visuo-constructional functions			
Rey–Osterrieth Complex Figure test (ROCF) – Copy	0.167	0.779	0.159

Significant *p*-value are reported in bold.

### 3.3 Long-term beneficial effects of face-to-face cognitive VRRS during anodal tDCS followed by cognitive telerehabilitation

Consistent with our second aim, we assessed the possibility of prolonging the beneficial effects obtained after FTF treatment with a beta regression analysis.

Regarding the neuropsychological assessment, as reported earlier, the outcome that behaved in a significantly different way in the three groups over time was the IFR of the FCSRT.

The analyses showed that the gains in episodic memory (FCSRT, IFR score) were maintained up to the 7-month (T3) follow-up only in the clinic-atDCS-VRRS+Tele@H-VRRS group. Specifically, the comparison between the effects of clinic-based ptDCS-VRRS combined with Tele@H-VRRS and clinic-based atDCS-VRRS combined with Tele@H-VRRS revealed an estimated effect of −0.5887 for clinic-ptDCS-VRRS+Tele@H-VRRS compared to clinic-atDCS-VRRS+Tele@H-VRRS (*p* = 0.047; odds ratio = 0.56) when clinic-atDCS-VRRS+Tele@H-VRRS was used as the reference group. Moreover, clinic-VRRS+Tele@H-VRRS had an estimated effect of −0.53 compared to that of clinic-atDCS-VRRS+Tele@H-VRRS (p = 0.06; odds ratio = 0.59), using clinic-atDCS-VRRS+Tele@H-VRRS as the reference group (Figure 3B). Additionally, the model showed a pseudo R-squared value of 0.088, explaining approximately 8.8% of the variability in the outcome variable.

### 3.4 System usability scale

Interestingly, the SUS, administered at T1 to the four groups who received clinic-VRRS, showed good usability performance of the clinic-VRRS system (72.2, SD 11.6), and the SUS scores obtained at T2 in the three groups that underwent home-based treatment (Tele@H-VRRS and @H-UCS) from T1 and T2 highlighted good usability performance (74.4, SD 9.4) of the VRRS telerehabilitation system (Bangor et al., 2008).

### 3.5 tDCS-perceptual sensations questionnaire

tDCS perceptual sensation questionnaire scores reported during anodal tDCS were compared with those reported during placebo tDCS (clinic-atDCS-VRRS+Tele@H-VRRS vs. clinic-ptDCS-VRRS+Tele@H-VRRS group), showing comparable tDCS-induced sensations in the two stimulation conditions (clinic-atDCS-VRRS+Tele@H-VRRS: 1.39 SD 0.90, clinic-ptDCS-VRRS+Tele@H-VRRS: 1.69 SD 0.98, U = 172.5, *p* = 0.230). Overall, only a few subjects reported low-intensity perceptual sensations related to the application of tDCS (burning, itching, and tingling).

## 4 Discussion

Episodic memory refers to the memory of past life events (Tulving, 1983) and displays the greatest degree of age-related decline (Rönnlund et al., 2005; Salthouse, 2011; Vestergren and Nilsson, 2011), a process that is accelerated in pathological conditions such as MCI and AD.

Non-pharmacological interventions to prevent and treat cognitive deficits and the associated difficulties with activities of daily living in neurodegenerative disease patients have gained attention in recent years. Among these interventions, cognitive training offers a potential approach for dementia prevention and for the improvement of cognitive functions (Cappa et al., 2003; Cotelli et al., 2006; Bahar-Fuchs et al., 2013a, 2013b; Yao et al., 2020; Hu et al., 2022). A critical aspect of cognitive training programs is that the most promising interventions involve intensive in-person sessions that are unlikely to be cost-effective or feasible for large-scale implementation (Botsis et al., 2008; Brennan et al., 2009; Corbetta et al., 2015). Within the framework of non-pharmacological interventions, the use of technology to assist people at risk of developing cognitive disorders or mild dementia at home has gradually gained importance (Realdon et al., 2016; Rossetto et al., 2023). Moreover, in recent years, tDCS has been considered a promising, noninvasive neuromodulation technique for individuals suffering from MCI (Palimariciuc et al., 2023). Since some evidence suggests that it might be worth exploring new cognitive rehabilitation approaches, we applied cognitive rehabilitation training combined with tDCS and telerehabilitation herein (Isernia et al., 2019; Nousia et al., 2021; Menengi̇ç et al., 2022; Torpil et al., 2023).

In particular, we evaluated the efficacy of cognitive virtual reality rehabilitation system (VRRS) combined with anodal transcranial direct current stimulation (tDCS) applied to the left dorsolateral prefrontal cortex (DLPFC) compared to that of placebo tDCS combined with VRRS, and we assessed the possibility of prolonging the beneficial effects. Referring to long-term memory, we targeted the left DLPFC, in line with previous studies that used noninvasive brain stimulation techniques to prove the involvement of this area in memory abilities (Fletcher and Henson, 2001; Rossi et al., 2001, 2004, 2006; Sandrini et al., 2003, 2013, 2014, 2016, 2019, 2020; Manenti et al., 2010, 2011, 2012, 2013, 2016, 2017, 2020b; Sandrini and Cohen, 2014; Brambilla et al., 2015; Krebs et al., 2020; Vaqué-Alcázar et al., 2021). Some researchers have used multiple sessions of tDCS to induce long-lasting effects in subjects with MCI (Yun et al., 2016; Fileccia et al., 2019; Gomes et al., 2019; Sandrini et al., 2020; Gu et al., 2022), but long-term effects of the intervention have been recorded in few studies (Murugaraja et al., 2017; Das et al., 2019; Gomes et al., 2019; Lu et al., 2019; Gu et al., 2022). At the neuronal level, in subjects with MCI, tDCS has been shown to increase regional cerebral metabolism in multiple brain regions, including the insula, hippocampus, and parahippocampus (Yun et al., 2016; Fregni et al., 2021).

Here, we found that an innovative in-person cognitive neurorehabilitation approach (FTF VRRS during anodal tDCS - clinic-atDCS-VRRS) was able to enhance episodic memory in MCI patients. The rationale for the application of tDCS in MCI and AD patients is based on modulating cortical excitability with a combined approach, which involves the induction of neuroplasticity through the activation of impaired cognitive functions in conjunction with tDCS. Therefore, the combined application of specific cognitive training and tDCS is essential for inducing synaptic plasticity mechanisms (Zimerman et al., 2013; Hsu et al., 2015; Prehn and Flöel, 2015; Yun et al., 2016; Gu et al., 2022).

In addition, we observed that MCI subjects who had received face-to-face cognitive rehabilitation combined with neuromodulation followed by asynchronous tablet telerehabilitation treatment (clinic-atDCS-VRRS+Tele@H-VRRS) maintained improvements in memory at the 7-month follow-up. Telerehabilitation technologies allow services to be provided remotely in patients’ homes, allowing access to health care to individuals living in rural settings or with mobility difficulties (Rogante et al., 2010; Brennan et al., 2011; Peretti et al., 2017; De Cola et al., 2020; Lawson et al., 2020; Maresca et al., 2020; Cruse et al., 2022). In addition, the telerehabilitation modality offers the advantage of providing rehabilitation within the natural environment of the patient’s home, making the treatment more realistic and possibly more generalizable to the person’s daily life (McCue et al., 2010).

In line with our previous results, this study confirms the feasibility of telerehabilitation in subjects with MCI, which is likely related to participants engagement in a telerehabilitation design involving asynchronous researcher–patient interactions (Matamala-Gomez et al., 2020). Overall, high rates of participant agreement, recruitment, and treatment adherence supported the feasibility of both in-person cognitive neurorehabilitation treatment (clinic-atDCS-VRRS) and telerehabilitation with home-based cognitive VRRS interventions (Tele@H-VRRS). Moreover, the analyses of system usability demonstrated the good usability of the VRRS applied in clinic (clinic-VRRS) and at home (Tele@H-VRRS). The present results are in line with recent findings and meta-analyses published on the efficacy and feasibility of a cognitive telerehabilitation program in individuals with MCI (Poon et al., 2005; Vermeij et al., 2016; Burton and O’Connell, 2018; Mosca et al., 2020; Nousia et al., 2021; Cacciante et al., 2022; Bernini et al., 2023; Chan et al., 2024).

In particular, MCI patients who received VRRS cognitive treatment in the clinic associated with anodal tDCS followed by VRRS tablet telerehabilitation showed greater maintenance of treatment gains in Immediate Free Recall (IFR) on the Free and Cued Selective Reminding Test (FCSRT), and these enhancements were maintained over a 7-month follow-up. The FCSRT (Buschke, 1984; Grober et al., 1997) is the memory test recommended by the International Working Group on AD (Dubois et al., 2007) for the detection of significant and progressive episodic memory impairment (Lemos et al., 2015). The FCSRT assesses verbal episodic memory with controlled learning and semantic cueing. This test has been shown to be useful in predicting the presence of dementia (Grober et al., 2000), in distinguishing AD from other dementias (Pillon et al., 1994; Pasquier et al., 2001) and in predicting the progression from MCI to AD (Sarazin et al., 2007; Frasson et al., 2011; Clerici et al., 2017). Interestingly, FCSRT scores have been shown to correlate with structural measures of hippocampal atrophy (Sarazin et al., 2010). Specifically, the treatment proposed in this paper involving the combination of anodal tDCS and cognitive training followed by TR improved the IFR of the FCRST in MCI patients, and this increase remained stable at the 7-month follow-up. While the present findings emphasize the importance of telerehabilitation for treating cognitive deficits to slow the progression of the disease, standardization of methodological aspects of the studies is required to obtain more homogenous data and to determine the optimal type and dose of cognitive telerehabilitation (Maggio et al., 2024).

We acknowledge that our study has some limitations. First, a larger sample size might allow us to account for individual differences that could influence the efficacy of the treatment; consequently, the findings should be confirmed in larger samples. Furthermore, in future trials, we will consider a cost-effectiveness analysis of the telerehabilitation approach (Dávalos et al., 2009) and the possibility of planning longer follow-up visits to follow the progress of the improvement obtained after treatment over a longer period of time. Moreover, the lack of control conditions applying tDCS over different cortical areas might represent further limitations of the present study. Furthermore, the placement of the reference electrode in a cephalic region (either anode or cathode) with an equally sized anodal electrode can induce reference-specific effects (anodal/ cathodal) in parallel to the cathodal/anodal effects of the active electrode. Finally, further studies could test for possible learning effects due to the repetition of the tests at several time points, even if the specificity of the results on episodic memory and the selective recording of improvements in the FTF VRRS during anodal tDCS (clinic-atDCS-VRRS) effect, recorded in only one group of subjects, suggest that the cognitive improvements observed in our study cannot be solely accounted for by task practice effects.

Nonetheless, the findings in this study are encouraging, providing preliminary evidence in support of individualized VRRS treatment coupled with tDCS and telerehabilitation for cognitive rehabilitation. Our results should pave the way for future studies aimed at identifying optimal treatment protocols for individuals with MCI. The combination of innovative technologies such as telerehabilitation and transcranial direct current stimulation may be particularly relevant for obtaining the best possible enhancement in subjects with limited access to therapy due to geographical distance, transport difficulties or a lack of local services.

In conclusion, although further research is needed, there is promising evidence for the implementation of transcranial current stimulation and telerehabilitation components in cognitive rehabilitation programs dedicated to individuals with MCI (Cotelli et al., 2019; Cacciante et al., 2022). Further studies are needed on the organizational aspects of TR service delivery, reimbursement for remotely delivered services, and ways to provide training for the involved health care personnel. A further development is the delivery of neurorehabilitation programs using noninvasive brain stimulation technology at home (Charvet et al., 2015, 2020; Pilloni et al., 2022).

## Data availability statement

The raw data supporting the conclusions of this article will be made available by the authors, without undue reservation.

## Ethics statement

The studies involving humans were approved by the local Ethics Committees. The studies were conducted in accordance with the local legislation and institutional requirements. The participants provided their written informed consent to participate in this study.

## Author contributions

RM: Methodology, Conceptualization, Data curation, Investigation, Writing – original draft, Writing – review & editing. FB: Methodology, Conceptualization, Data curation, Investigation, Writing – review & editing. IP: Data curation, Investigation, Writing – original draft, Writing – review & editing. EG: Data curation, Investigation, Writing – original draft, Writing – review & editing. EC: Data curation, Investigation, Writing – original draft, Writing – review & editing. CA: Data curation, Investigation, Writing – review & editing. FR: Data curation, Investigation, Writing – review & editing. ST: Data curation, Investigation, Writing – review & editing. CP: Data curation, Investigation, Writing – review & editing. AG: Formal analysis, Writing – original draft, Writing – review & editing. NSB: Formal analysis, Writing – original draft, Writing – review & editing. RC: Data curation, Investigation, Writing – review & editing. VC: Investigation, Writing – review & editing. GB: Investigation, Writing – review & editing. AQ: Conceptualization, Methodology, Investigation, Writing – review & editing. PB: Conceptualization, Methodology, Investigation, Writing – review & editing. SC: Conceptualization, Methodology, Investigation, Writing – review & editing. PR: Investigation, Writing – review & editing. MC: Data curation, Investigation, Conceptualization, Methodology, Writing – original draft, Writing – review & editing.

## Footnotes

^1^ http://khymeia.com/

^2^Soterix Medical; https://soterixmedical.com.

^3^ https://training.cochrane.org/handbook/current/chapter-08

## Glossary

@H-UCS: at Home Unstructured Cognitive Stimulation
Δ: deltaT
AD: Alzheimer’s Disease
aMCI-m: amnestic multiple-domain Mild Cognitive Impairment
aMCI-s: amnestic single-domain Mild Cognitive Impairment
atDCS: anodal transcranial Direct Current Stimulation
AR1: first order autoregressive
BADA: Battery for Analysis of Aphasic Deficit
BADL: Basic Activity of Daily Living
CDR: Clinical Dementia Rating
clinic-atDCS-VRRS: face-to-face cognitive Virtual Reality Rehabilitation System during anodal transcranial Direct Current Stimulation
clinic-atDCS-VRRS+Tele@H-VRRS: face-to-face cognitive Virtual Reality Rehabilitation System during anodal transcranial Direct Current Stimulation followed by cognitive TeleRehabilitation
clinic-ptDCS-VRRS: face-to-face cognitive Virtual Reality Rehabilitation System during placebo transcranial Direct Current Stimulation;
clinic-ptDCS-VRRS+Tele@H-VRRS: face-to-face cognitive Virtual Reality Rehabilitation System during placebo transcranial Direct Current Stimulation followed by cognitive TeleRehabilitation
clinic-TAU: face-to-face cognitive Treatment As Usual
clinic-VRRS: face-to-face cognitive Virtual Reality Rehabilitation System
clinic-VRRS+@H-UCS: face-to-face cognitive Virtual Reality Rehabilitation System followed by at-home Unstructured Cognitive Stimulation
clinic-VRRS+Tele@H-VRRS: face-to-face cognitive Virtual Reality Rehabilitation System followed by cognitive TeleRehabilitation
cm: centimeters
CNDs: Chronic Neurological Disorders
CRI-q: Cognitive Reserve Index questionnaire
DALY: Disability-Adjusted Life-Years
DLPFC: DorsoLateral PreFrontal Cortex
DFR: Delayed Free Recall
DSM: Diagnostic and Statistical Manual of Mental Disorders
DTR: Delayed Total Recall
EEG: ElectroEncephaloGraphy
EHI: Edinburgh Handedness Inventory
EMQ: Everyday Memory Questionnaire
FCSRT: Free and Cued Selective Reminding Test
FTF: Face-To-Face
GBD: Global Burden of Disease
GDS: Geriatric Depression Scale
GLMMs: Generalized Linear Mixed Models
HALE: Healthy Life Expectancy
IADL: Instrumental Activity of Daily Living
IFR: immediate Free Recall
ISC: Index of Sensitivity of Cueing
ITR: Immediate Total Recall
mA: milliAmpere
MCI: Mild Cognitive Impairment
Min: minutes
mITT: modified Intention To Treat
MMSE: Mini Mental State Examination
naMCI-m: nonamnestic multiple-domain Mild Cognitive Impairment
naMCI-s: nonamnestic single-domain Mild Cognitive Impairment
NPI: NeuroPsychiatric Inventory
ptDCS: placebo transcranial Direct Current Stimulation
QOL-AD: Quality of Life in Alzheimer’s Disease
RAVLT: Rey Auditory Verbal Learning Test
RCT: Randomized Controlled Trial
ROCF: Rey–Osterrieth Complex Figure
SD: Standard Deviation
S: seconds
SPSS: Statistical Package for Social Science
SUS: System Usability Scale
T: Timepoint
T0: baseline Timepoint
T1: post-treatment Timepoint
T2: 4 months from baseline Timepoint
T3: 7 months from baseline Timepoint
TAU: Treatment As Usual
tDCS: transcranial Direct Current Stimulation
Tele@H-VRRS: at Home Virtual Reality Rehabilitation System
TMT: Trail Making Test
TR: TeleRehabilitation
VRRS: Virtual Reality Rehabilitation System

## References

[ref1] Abraha I. Montedori A. (2010). Modified intention to treat reporting in randomised controlled trials: systematic review. BMJ 340:c2697. doi: 10.1136/bmj.c2697 20547685 PMC2885592

[ref2] Alaimo C. Campana E. Stoppelli M. R. Gobbi E. Baglio F. Rossetto F.. (2021). Cognitive tele-enhancement in healthy older adults and subjects with subjective memory complaints: a review. Front. Neurol. 12:650553. doi: 10.3389/fneur.2021.650553, PMID: 34290660 PMC8287022

[ref3] American Psychiatric Association (2014). Manuale diagnostico e statistico dei disturbi mentali. Milano: Raffaele Cortina Editore.

[ref4] Antal A. Alekseichuk I. Bikson M. Brockmöller J. Brunoni A. R. Chen R.. (2017). Low intensity transcranial electric stimulation: safety, ethical, legal regulatory and application guidelines. Clin. Neurophysiol. 128, 1774–1809. doi: 10.1016/j.clinph.2017.06.001, PMID: 28709880 PMC5985830

[ref5] Antonietti A. Gandolla M. Rossini M. Molteni F. Pedrocchi A. Consortium A. (2017). Interference between cognitive and motor recovery in elderly dementia patients through a holistic tele-rehabilitation platform. In: *Wireless Mobile Communication and Healthcare: 6th International Conference, MobiHealth 2016, Milan, Italy, November 14–16, 2016, Proceedings 6*: Springer, pp. 359–366.

[ref6] Astell A. J. Bouranis N. Hoey J. Lindauer A. Mihailidis A. Nugent C.. (2019). Technology and dementia: the future is now. Dement. Geriatr. Cogn. Disord. 47, 131–139. doi: 10.1159/000497800, PMID: 31247624 PMC6643496

[ref7] Bahar-Fuchs A. Clare L. Woods B. (2013a). Cognitive training and cognitive rehabilitation for mild to moderate Alzheimer's disease and vascular dementia. Cochrane Database Syst. Rev. 2013:Cd003260. doi: 10.1002/14651858.CD003260.pub2, PMID: 23740535 PMC7144738

[ref8] Bahar-Fuchs A. Clare L. Woods B. (2013b). Cognitive training and cognitive rehabilitation for persons with mild to moderate dementia of the Alzheimer's or vascular type: a review. J Alzheimers Res. Ther. 5, 35–14. doi: 10.1186/alzrt189, PMID: 23924584 PMC3979126

[ref9] Bahar-Fuchs A. Martyr A. Goh A. M. Sabates J. Clare L. (2019). Cognitive training for people with mild to moderate dementia. Cochrane Database Syst. Rev. 3:Cd013069. doi: 10.1002/14651858.CD013069.pub2, PMID: 30909318 PMC6433473

[ref10] Bangor A. Kortum P. T. Miller J. T. (2008). An empirical evaluation of the system usability scale. Int. J. Hum. Comput. Interact. 24, 574–594. doi: 10.1080/10447310802205776

[ref11] Bangor A. Kortum P. Miller J. (2009). Determining what individual SUS scores mean: adding an adjective rating scale. J. Usability Stud. 4, 114–123.

[ref12] Basso A. Capitani E. Laiacona M. (1987). Raven's coloured progressive matrices: normative values on 305 adult normal controls. Funct. Neurol. 2, 189–194, PMID: 3666548

[ref13] Bernini S. Panzarasa S. Quaglini S. Costa A. Picascia M. Cappa S. F.. (2023). HomeCoRe system for telerehabilitation in individuals at risk of dementia: a usability and user experience study. Front. Med. (Lausanne) 10:1129914. doi: 10.3389/fmed.2023.1129914, PMID: 36873886 PMC9983032

[ref14] Bharucha A. J. Anand V. Forlizzi J. Dew M. A. Reynolds C. F. 3rd Stevens S.. (2009). Intelligent assistive technology applications to dementia care: current capabilities, limitations, and future challenges. Am. J. Geriatr. Psychiatry 17, 88–104. doi: 10.1097/JGP.0b013e318187dde5, PMID: 18849532 PMC2768007

[ref15] Bianchetti A. Cornali C. Ranieri P. Trabucchi M. (2017). Quality of life in patients with mild dementia. Validation of the Italian version of the quality of life Alzheimer’s disease (QoL-AD) scale. Official J. Ital. Soc. Gerontol. Geriatr. 137:11.

[ref16] Bikson M. Grossman P. Thomas C. Zannou A. L. Jiang J. Adnan T.. (2016). Safety of transcranial direct current stimulation: evidence based update 2016. Brain Stimul. 9, 641–661. doi: 10.1016/j.brs.2016.06.004, PMID: 27372845 PMC5007190

[ref17] Binetti G. Mega M. S. Magni E. Padovani A. Rozzini L. Bianchetti A.. (1998). Behavioral disorders in Alzheimer disease: a transcultural perspective. Arch. Neurol. 55, 539–544. doi: 10.1001/archneur.55.4.539, PMID: 9561983

[ref18] Botsis T. Demiris G. Pedersen S. Hartvigsen G. (2008). Home telecare technologies for the elderly. J. Telemed. Telecare 14, 333–337. doi: 10.1258/jtt.2008.007002 18852311

[ref19] Boutron I. Altman D. G. Moher D. Schulz K. F. Ravaud P. (2017). CONSORT statement for randomized trials of nonpharmacologic treatments: a 2017 update and a CONSORT extension for nonpharmacologic trial abstracts. Ann. Intern. Med. 167, 40–47. doi: 10.7326/m17-0046, PMID: 28630973

[ref20] Boutron I. Moher D. Altman D. G. Schulz K. F. Ravaud P. (2008). Extending the CONSORT statement to randomized trials of nonpharmacologic treatment: explanation and elaboration. Ann. Intern. Med. 148, 295–309. doi: 10.7326/0003-4819-148-4-200802190-00008, PMID: 18283207

[ref21] Brambilla M. Manenti R. Ferrari C. Cotelli M. (2015). Better together: left and right hemisphere engagement to reduce age-related memory loss. Behav. Brain Res. 293, 125–133. doi: 10.1016/j.bbr.2015.07.037, PMID: 26200716

[ref22] Brem A. K. Di Iorio R. Fried P. J. Oliveira-Maia A. J. Marra C. Profice P.. (2020). Corticomotor plasticity predicts clinical efficacy of combined Neuromodulation and cognitive training in Alzheimer's disease. Front. Aging Neurosci. 12:200. doi: 10.3389/fnagi.2020.00200, PMID: 32733232 PMC7360860

[ref23] Brennan D. Georgeadis A. Baron C. (2002). Telerehabilitation tools for the provision of remote speech-language treatment. Top. Stroke Rehabil. 8, 71–78. doi: 10.1310/u7kv-dy7u-q6qp-lvbp, PMID: 14523731

[ref24] Brennan D. M. Mawson S. Brownsell S. (2009). Telerehabilitation: enabling the remote delivery of healthcare, rehabilitation, and self management. Stud. Health Technol. Inform. 145, 231–248. doi: 10.3233/978-1-60750-018-6-231, PMID: 19592797

[ref25] Brennan D. M. Tindall L. Theodoros D. Brown J. Campbell M. Christiana D.. (2011). A blueprint for telerehabilitation guidelines—October 2010. J. Telemed. E Health 17, 662–665. doi: 10.1089/tmj.2011.0036, PMID: 21790271

[ref26] Brooke J. (1996). “SUS-A quick and dirty usability scale” in Usability evaluation in industry. ed. Brooke J., vol. 189 (Milton Park: Taylor and Francis), 4–7.

[ref27] Brunoni A. R. Nitsche M. A. Bolognini N. Bikson M. Wagner T. Merabet L.. (2012). Clinical research with transcranial direct current stimulation (tDCS): challenges and future directions. Brain Stimul. 5, 175–195. doi: 10.1016/j.brs.2011.03.002, PMID: 22037126 PMC3270156

[ref28] Burton R. L. O'Connell M. E. (2018). Telehealth rehabilitation for cognitive impairment: randomized controlled feasibility trial. JMIR Res. Protoc. 7:e43. doi: 10.2196/resprot.9420, PMID: 29422453 PMC5824099

[ref29] Buschke H. (1984). Cued recall in amnesia. J. Clin. Exp. Neuropsychol. 6, 433–440. doi: 10.1080/01688638408401233 6501581

[ref30] Cacciante L. Pietà C. D. Rutkowski S. Cieślik B. Szczepańska-Gieracha J. Agostini M.. (2022). Cognitive telerehabilitation in neurological patients: systematic review and meta-analysis. Neurol. Sci. 43, 847–862. doi: 10.1007/s10072-021-05770-6, PMID: 34822030 PMC8613517

[ref31] Caffarra P. Vezzadini G. Dieci F. Zonato F. Venneri A. (2002). Rey-Osterrieth complex figure: normative values in an Italian population sample. Neurol. Sci. 22, 443–447. doi: 10.1007/s100720200003, PMID: 11976975

[ref32] Calabria M. Manenti R. Rosini S. Zanetti O. Miniussi C. Cotelli M. (2011). Objective and subjective memory impairment in elderly adults: a revised version of the everyday memory questionnaire. Aging Clin. Exp. Res. 23, 67–73. doi: 10.1007/bf03324954, PMID: 21499021

[ref33] Cappa S. F. Benke T. Clarke S. Rossi B. Stemmer B. van Heugten C. M. (2003). EFNS guidelines on cognitive rehabilitation: report of an EFNS task force. Eur. J. Neurol. 10, 11–23. doi: 10.1046/j.1468-1331.2003.00537.x, PMID: 12534988

[ref34] Cappa S. F. Benke T. Clarke S. Rossi B. Stemmer B. van Heugten C. M. (2005). EFNS guidelines on cognitive rehabilitation: report of an EFNS task force. Eur. J. Neurol. 12, 665–680. doi: 10.1111/j.1468-1331.2005.01330.x, PMID: 16128867

[ref35] Carlesimo G. A. Caltagirone C. Gainotti G. (1996). The mental deterioration battery: normative data, diagnostic reliability and qualitative analyses of cognitive impairment. The Group for the Standardization of the mental deterioration battery. Eur. Neurol. 36, 378–384. doi: 10.1159/000117297, PMID: 8954307

[ref36] Chan A. T. C. Ip R. T. F. Tran J. Y. S. Chan J. Y. C. Tsoi K. K. F. (2024). Computerized cognitive training for memory functions in mild cognitive impairment or dementia: a systematic review and meta-analysis. NPJ Digit. Med. 7:1. doi: 10.1038/s41746-023-00987-5 38172429 PMC10764827

[ref37] Charvet L. E. Kasschau M. Datta A. Knotkova H. Stevens M. C. Alonzo A.. (2015). Remotely-supervised transcranial direct current stimulation (tDCS) for clinical trials: guidelines for technology and protocols. Front. Syst. Neurosci. 9:26. doi: 10.3389/fnsys.2015.00026, PMID: 25852494 PMC4362220

[ref38] Charvet L. E. Shaw M. T. Bikson M. Woods A. J. Knotkova H. (2020). Supervised transcranial direct current stimulation (tDCS) at home: a guide for clinical research and practice. Brain Stimul. 13, 686–693. doi: 10.1016/j.brs.2020.02.011, PMID: 32289698

[ref39] Cherney L. R. van Vuuren S. (2012). Telerehabilitation, virtual therapists, and acquired neurologic speech and language disorders. Semin. Speech Lang. 33, 243–257. doi: 10.1055/s-0032-1320044, PMID: 22851346 PMC3691350

[ref40] Cieza A. Causey K. Kamenov K. Hanson S. W. Chatterji S. Vos T. (2021). Global estimates of the need for rehabilitation based on the global burden of disease study 2019: a systematic analysis for the global burden of disease study 2019. Lancet 396, 2006–2017. doi: 10.1016/s0140-6736(20)32340-0, PMID: 33275908 PMC7811204

[ref41] Clare L. (2017). Rehabilitation for people living with dementia: a practical framework of positive support. PLoS Med. 14:e1002245. doi: 10.1371/journal.pmed.1002245, PMID: 28267744 PMC5340348

[ref42] Clare L. Linden D. E. Woods R. T. Whitaker R. Evans S. J. Parkinson C. H.. (2010). Goal-oriented cognitive rehabilitation for people with early-stage Alzheimer disease: a single-blind randomized controlled trial of clinical efficacy. Am. J. Geriatr. Psychiatry 18, 928–939. doi: 10.1097/JGP.0b013e3181d5792a, PMID: 20808145

[ref43] Clare L. Woods R. T. (2004). Cognitive training and cognitive rehabilitation for people with early-stage Alzheimer's disease: a review. J. Neuropsychol. Rehabil. 14, 385–401. doi: 10.1080/09602010443000074

[ref44] Clerici F. Ghiretti R. Di Pucchio A. Pomati S. Cucumo V. Marcone A.. (2017). Construct validity of the free and cued selective reminding test in older adults with memory complaints. J. Neuropsychol. 11, 238–251. doi: 10.1111/jnp.12087, PMID: 26560406

[ref45] Corbett A. Owen A. Hampshire A. Grahn J. Stenton R. Dajani S.. (2015). The effect of an online cognitive training package in healthy older adults: an online randomized controlled trial. J. Am. Med. Dir. Assoc. 16, 990–997. doi: 10.1016/j.jamda.2015.06.014, PMID: 26543007

[ref46] Corbetta D. Imeri F. Gatti R. (2015). Rehabilitation that incorporates virtual reality is more effective than standard rehabilitation for improving walking speed, balance and mobility after stroke: a systematic review. J. Physiother. 61, 117–124. doi: 10.1016/j.jphys.2015.05.017, PMID: 26093805

[ref47] Cotelli M. Calabria M. Zanetti O. (2006). Cognitive rehabilitation in Alzheimer's disease. Aging Clin. Exp. Res. 18, 141–143. doi: 10.1007/bf03327429 16702783

[ref48] Cotelli M. Manenti R. Alberici A. Brambilla M. Cosseddu M. Zanetti O.. (2012). Prefrontal cortex rTMS enhances action naming in progressive non-fluent aphasia. Eur. J. Neurol. 19, 1404–1412. doi: 10.1111/j.1468-1331.2012.03699.x, PMID: 22435956

[ref49] Cotelli M. Manenti R. Brambilla M. Gobbi E. Ferrari C. Binetti G.. (2019). Cognitive telerehabilitation in mild cognitive impairment, Alzheimer's disease and frontotemporal dementia: a systematic review. J. Telemed. Telecare 25, 67–79. doi: 10.1177/1357633x17740390, PMID: 29117794

[ref50] Cotelli M. Manenti R. Ferrari C. Gobbi E. Macis A. Cappa S. F. (2020). Effectiveness of language training and non-invasive brain stimulation on oral and written naming performance in primary progressive aphasia: a meta-analysis and systematic review. Neurosci. Biobehav. Rev. 108, 498–525. doi: 10.1016/j.neubiorev.2019.12.003, PMID: 31811834

[ref51] Cruse N. Piotto V. Coelho C. Behn N. (2022). Telehealth administration of narrative and procedural discourse: a UK and US comparison of traumatic brain injury and matched controls. Int. J. Lang. Commun. Disord. 59, 519–531. doi: 10.1111/1460-6984.12813, PMID: 36377239

[ref52] Cummings J. L. Mega M. Gray K. Rosenberg-Thompson S. Carusi D. A. Gornbein J. (1994). The neuropsychiatric inventory: comprehensive assessment of psychopathology in dementia. Neurology 44, 2308–2314. doi: 10.1212/wnl.44.12.2308 7991117

[ref53] Das N. Spence J. S. Aslan S. Vanneste S. Mudar R. Rackley A.. (2019). Cognitive training and transcranial direct current stimulation in mild cognitive impairment: a randomized pilot trial. Front. Neurosci. 13:307. doi: 10.3389/fnins.2019.00307, PMID: 31031581 PMC6473050

[ref54] Dávalos M. E. French M. T. Burdick A. E. Simmons S. C. (2009). Economic evaluation of telemedicine: review of the literature and research guidelines for benefit-cost analysis. Telemed. J. E Health 15, 933–948. doi: 10.1089/tmj.2009.0067 19954346

[ref55] Dayan E. Censor N. Buch E. R. Sandrini M. Cohen L. G. (2013). Noninvasive brain stimulation: from physiology to network dynamics and back. Nat. Neurosci. 16, 838–844. doi: 10.1038/nn.3422, PMID: 23799477 PMC4876726

[ref56] De Cola M. C. Maresca G. D'Aleo G. Carnazza L. Giliberto S. Maggio M. G.. (2020). Teleassistance for frail elderly people: a usability and customer satisfaction study. Geriatr. Nurs. 41, 463–467. doi: 10.1016/j.gerinurse.2020.01.019, PMID: 32067831

[ref57] Dubois B. Feldman H. H. Jacova C. DeKosky S. T. Barberger-Gateau P. Cummings J.. (2007). Research criteria for the diagnosis of Alzheimer's disease: revising the NINCDS–ADRDA criteria. Lancet Neurol. 6, 734–746. doi: 10.1016/S1474-4422(07)70178-3, PMID: 17616482

[ref58] Fertonani A. Ferrari C. Miniussi C. (2015). What do you feel if I apply transcranial electric stimulation? Safety, sensations and secondary induced effects. Clin. Neurophysiol. 126, 2181–2188. doi: 10.1016/j.clinph.2015.03.015, PMID: 25922128

[ref59] Fileccia E. Di Stasi V. Poda R. Rizzo G. Stanzani-Maserati M. Oppi F.. (2019). Effects on cognition of 20-day anodal transcranial direct current stimulation over the left dorsolateral prefrontal cortex in patients affected by mild cognitive impairment: a case-control study. Neurol. Sci. 40, 1865–1872. doi: 10.1007/s10072-019-03903-6, PMID: 31062189

[ref60] Fletcher P. C. Henson R. N. (2001). Frontal lobes and human memory: insights from functional neuroimaging. Brain 124, 849–881. doi: 10.1093/brain/124.5.849 11335690

[ref61] Folstein M. F. Folstein S. E. McHugh P. R. (1975). “Mini-mental state”: a practical method for grading the cognitive state of patients for the clinician. J. Psychiatr. Res. 12, 189–198. doi: 10.1016/0022-3956(75)90026-6 1202204

[ref62] Frasson P. Ghiretti R. Catricalà E. Pomati S. Marcone A. Parisi L.. (2011). Free and cued selective reminding test: an Italian normative study. Neurol. Sci. 32, 1057–1062. doi: 10.1007/s10072-011-0607-3, PMID: 21594655

[ref63] Fregni F. El-Hagrassy M. M. Pacheco-Barrios K. Carvalho S. Leite J. Simis M.. (2021). Evidence-based guidelines and secondary Meta-analysis for the use of transcranial direct current stimulation in neurological and psychiatric disorders. Int. J. Neuropsychopharmacol. 24, 256–313. doi: 10.1093/ijnp/pyaa051, PMID: 32710772 PMC8059493

[ref64] Frisoni G. B. Altomare D. Ribaldi F. Villain N. Brayne C. Mukadam N.. (2023). Dementia prevention in memory clinics: recommendations from the European task force for brain health services. Lancet Reg. Health Eur. 26:100576. doi: 10.1016/j.lanepe.2022.100576, PMID: 36895446 PMC9989648

[ref65] Gates N. J. Sachdev P. (2014). Is cognitive training an effective treatment for preclinical and early Alzheimer's disease? J. Alzheimers Dis. 42, S551–S559. doi: 10.3233/jad-141302, PMID: 25171716

[ref66] GBD 2017 DALYs and HALE Collaborators (2018). Global, regional, and national disability-adjusted life-years (DALYs) for 359 diseases and injuries and healthy life expectancy (HALE) for 195 countries and territories, 1990-2017: a systematic analysis for the global burden of disease study 2017. Lancet 392, 1859–1922. doi: 10.1016/s0140-6736(18)32335-3, PMID: 30415748 PMC6252083

[ref67] GBD 2019 Diseases and Injuries Collaborators (2020). Global burden of 369 diseases and injuries in 204 countries and territories, 1990-2019: a systematic analysis for the global burden of disease study 2019. Lancet 396, 1204–1222. doi: 10.1016/s0140-6736(20)30925-9, PMID: 33069326 PMC7567026

[ref68] GBD 2021 Nervous System Disorders Collaborators (2024). Global, regional, and national burden of disorders affecting the nervous system, 1990-2021: a systematic analysis for the global burden of disease study 2021. Lancet Neurol. 23, 344–381. doi: 10.1016/S1474-4422(24)00038-3, PMID: 38493795 PMC10949203

[ref69] Giovagnoli A. R. Del Pesce M. Mascheroni S. Simoncelli M. Laiacona M. Capitani E. (1996). Trail making test: normative values from 287 normal adult controls. Ital. J. Neurol. Sci. 17, 305–309. doi: 10.1007/bf01997792, PMID: 8915764

[ref70] Gomes M. A. Akiba H. T. Gomes J. S. Trevizol A. P. de Lacerda A. L. T. Dias Á.. (2019). Transcranial direct current stimulation (tDCS) in elderly with mild cognitive impairment: a pilot study. Dement. Neuropsychol. 13, 187–195. doi: 10.1590/1980-57642018dn13-020007 31285793 PMC6601303

[ref71] Grober E. Lipton R. B. Hall C. Crystal H. (2000). Memory impairment on free and cued selective reminding predicts dementia. Neurology 54, 827–832. doi: 10.1212/wnl.54.4.827 10690971

[ref72] Grober E. Merling A. Heimlich T. Lipton R. B. (1997). Free and cued selective reminding and selective reminding in the elderly. J. Clin. Exp. Neuropsychol. 19, 643–654. doi: 10.1080/01688639708403750 9408795

[ref73] Gu J. Li D. Li Z. Guo Y. Qian F. Wang Y.. (2022). The effect and mechanism of transcranial direct current stimulation on episodic memory in patients with mild cognitive impairment. Front. Neurosci. 16:811403. doi: 10.3389/fnins.2022.811403, PMID: 35250453 PMC8891804

[ref74] Hong Y. J. Jang E. H. Hwang J. Roh J. H. Lee J. H. (2015). The efficacy of cognitive intervention programs for mild cognitive impairment: a systematic review. Curr. Alzheimer Res. 12, 527–542. doi: 10.2174/1567205012666150530201636 26027815

[ref75] Hsu W. Y. Ku Y. Zanto T. P. Gazzaley A. (2015). Effects of noninvasive brain stimulation on cognitive function in healthy aging and Alzheimer's disease: a systematic review and meta-analysis. Neurobiol. Aging 36, 2348–2359. doi: 10.1016/j.neurobiolaging.2015.04.016, PMID: 26022770 PMC4496249

[ref76] Hu M. Hu H. Shao Z. Gao Y. Zeng X. Shu X.. (2022). Effectiveness and acceptability of non-pharmacological interventions in people with mild cognitive impairment: overview of systematic reviews and network meta-analysis. J. Affect. Disord. 311, 383–390. doi: 10.1016/j.jad.2022.05.043, PMID: 35597472

[ref77] Isernia S. Di Tella S. Pagliari C. Jonsdottir J. Castiglioni C. Gindri P.. (2020). Effects of an innovative Telerehabilitation intervention for people with Parkinson's disease on quality of life, motor, and non-motor abilities. Front. Neurol. 11:846. doi: 10.3389/fneur.2020.00846, PMID: 32903506 PMC7438538

[ref78] Isernia S. Pagliari C. Jonsdottir J. Castiglioni C. Gindri P. Gramigna C.. (2019). Efficiency and patient-reported outcome measures from clinic to home: the human empowerment aging and disability program for digital-health rehabilitation. Front. Neurol. 10:1206. doi: 10.3389/fneur.2019.01206, PMID: 31824398 PMC6882300

[ref79] Jelcic N. Agostini M. Meneghello F. Bussè C. Parise S. Galano A.. (2014). Feasibility and efficacy of cognitive telerehabilitation in early Alzheimer's disease: a pilot study. Clin. Interv. Aging 9, 1605–1611. doi: 10.2147/cia.S68145, PMID: 25284993 PMC4181448

[ref80] Kairy D. Lehoux P. Vincent C. Visintin M. (2009). A systematic review of clinical outcomes, clinical process, healthcare utilization and costs associated with telerehabilitation. Disabil. Rehabil. 31, 427–447. doi: 10.1080/09638280802062553, PMID: 18720118

[ref81] Katz S. (1983). Assessing self-maintenance: activities of daily living, mobility, and instrumental activities of daily living. J. Am. Geriatr. Soc. 31, 721–727. doi: 10.1111/j.1532-5415.1983.tb03391.x, PMID: 6418786

[ref82] Kortte K. B. Rogalski E. J. (2013). Behavioural interventions for enhancing life participation in behavioural variant frontotemporal dementia and primary progressive aphasia. Int. Rev. Psychiatry 25, 237–245. doi: 10.3109/09540261.2012.751017, PMID: 23611353 PMC3659798

[ref83] Krebs C. Klöppel S. Heimbach B. Peter J. (2020). Education moderates the effect of tDCS on episodic memory performance in cognitively impaired patients. Brain Stimul. 13, 1396–1398. doi: 10.1016/j.brs.2020.07.008, PMID: 32712342

[ref84] Kudlicka A. Martyr A. Bahar-Fuchs A. Sabates J. Woods B. Clare L. (2023). Cognitive rehabilitation for people with mild to moderate dementia. Cochrane Database Syst. Rev. 2023:3388. doi: 10.1002/14651858.CD013388.pub2 PMC10310315 37389428

[ref85] Lawson D. W. Stolwyk R. J. Ponsford J. L. McKenzie D. P. Downing M. G. Wong D. (2020). Telehealth delivery of memory rehabilitation following stroke. J. Int. Neuropsychol. Soc. 26, 58–71. doi: 10.1017/s1355617719000651, PMID: 31983368

[ref86] Lawton M. Brody E. (1988). Instrumental activities of daily living (Iadl) scale-self-rated version. Psychopharmacol. Bull. 24, 789–791. 3249786

[ref87] Lefaucheur J. P. Antal A. Ayache S. S. Benninger D. H. Brunelin J. Cogiamanian F.. (2017). Evidence-based guidelines on the therapeutic use of transcranial direct current stimulation (tDCS). Clin. Neurophysiol. 128, 56–92. doi: 10.1016/j.clinph.2016.10.087, PMID: 27866120

[ref88] Lemos R. Simões M. R. Santiago B. Santana I. (2015). The free and cued selective reminding test: validation for mild cognitive impairment and Alzheimer's disease. J. Neuropsychol. 9, 242–257. doi: 10.1111/jnp.12048, PMID: 24894485

[ref89] Lezak M. Howieson D. Bigler E. Tranel D. (2012). Neuropsychological assessment. 5th Edn. Oxford: University Press.

[ref90] Livingston G. Sommerlad A. Orgeta V. Costafreda S. G. Huntley J. Ames D.. (2017). Dementia prevention, intervention, and care. Lancet 390, 2673–2734. doi: 10.1016/s0140-6736(17)31363-6 28735855

[ref91] Lu H. Chan S. S. M. Chan W. C. Lin C. Cheng C. P. W. Linda Chiu Wa L. (2019). Randomized controlled trial of TDCS on cognition in 201 seniors with mild neurocognitive disorder. Ann. Clin. Transl. Neurol. 6, 1938–1948. doi: 10.1002/acn3.50823, PMID: 31529691 PMC6801176

[ref92] Maggio M. G. Baglio F. Arcuri F. Borgnis F. Contrada M. Maldonado Diaz M. D.. (2024). Cognitive Telerehabilitation: an expert consensus paper on current evidence and future perspective. Front. Neurol. 15:1338873. doi: 10.3389/fneur.2024.1338873, PMID: 38426164 PMC10902044

[ref93] Maggio M. G. De Bartolo D. Calabrò R. S. Ciancarelli I. Cerasa A. Tonin P.. (2023). Computer-assisted cognitive rehabilitation in neurological patients: state-of-art and future perspectives. Front. Neurol. 14:1255319. doi: 10.3389/fneur.2023.1255319, PMID: 37854065 PMC10580980

[ref94] Manenti R. Brambilla M. Petesi M. Ferrari C. Cotelli M. (2013). Enhancing verbal episodic memory in older and young subjects after non-invasive brain stimulation. Front. Aging Neurosci. 5:49. doi: 10.3389/fnagi.2013.00049, PMID: 24062685 PMC3769624

[ref95] Manenti R. Cotelli M. Calabria M. Maioli C. Miniussi C. (2010). The role of the dorsolateral prefrontal cortex in retrieval from long-term memory depends on strategies: a repetitive transcranial magnetic stimulation study. Neuroscience 166, 501–507. doi: 10.1016/j.neuroscience.2009.12.037, PMID: 20034547

[ref96] Manenti R. Cotelli M. Miniussi C. (2011). Successful physiological aging and episodic memory: a brain stimulation study. Behav. Brain Res. 216, 153–158. doi: 10.1016/j.bbr.2010.07.027, PMID: 20667492

[ref97] Manenti R. Cotelli M. Robertson I. H. Miniussi C. (2012). Transcranial brain stimulation studies of episodic memory in young adults, elderly adults and individuals with memory dysfunction: a review. Brain Stimul. 5, 103–109. doi: 10.1016/j.brs.2012.03.004, PMID: 22503472

[ref98] Manenti R. Gobbi E. Baglio F. Macis A. Ferrari C. Pagnoni I.. (2020a). Effectiveness of an innovative cognitive treatment and Telerehabilitation on subjects with mild cognitive impairment: a multicenter, randomized, active-controlled study. Front. Aging Neurosci. 12:585988. doi: 10.3389/fnagi.2020.585988, PMID: 33304267 PMC7701275

[ref99] Manenti R. Sandrini M. Brambilla M. Cotelli M. (2016). The optimal timing of stimulation to induce long-lasting positive effects on episodic memory in physiological aging. Behav. Brain Res. 311, 81–86. doi: 10.1016/j.bbr.2016.05.028, PMID: 27185737

[ref100] Manenti R. Sandrini M. Gobbi E. Binetti G. Cotelli M. (2020b). Effects of transcranial direct current stimulation on episodic memory in amnestic mild cognitive impairment: a pilot study. J. Gerontol. B Psychol. Sci. Soc. Sci. 75, 1403–1413. doi: 10.1093/geronb/gby134, PMID: 30395314

[ref101] Manenti R. Sandrini M. Gobbi E. Cobelli C. Brambilla M. Binetti G.. (2017). Strengthening of existing episodic memories through non-invasive stimulation of prefrontal cortex in older adults with subjective memory complaints. Front. Aging Neurosci. 9:401. doi: 10.3389/fnagi.2017.00401, PMID: 29259554 PMC5723311

[ref102] Maresca G. Maggio M. G. De Luca R. Manuli A. Tonin P. Pignolo L.. (2020). Tele-neuro-rehabilitation in Italy: state of the art and future perspectives. Front. Neurol. 11:563375. doi: 10.3389/fneur.2020.563375, PMID: 33101176 PMC7554582

[ref103] Marion J. D. Althouse A. D. (2023). The use of historical controls in clinical trials. JAMA 330, 1484–1485. doi: 10.1001/jama.2023.16182 37768654

[ref104] Mashima P. A. Doarn C. R. (2008). Overview of telehealth activities in speech-language pathology. Telemed. J. E Health 14, 1101–1117. doi: 10.1089/tmj.2008.0080, PMID: 19119834

[ref105] Matamala-Gomez M. Maisto M. Montana J. I. Mavrodiev P. A. Baglio F. Rossetto F.. (2020). The role of engagement in Teleneurorehabilitation: a systematic review. Front. Neurol. 11:354. doi: 10.3389/fneur.2020.00354, PMID: 32435227 PMC7218051

[ref106] McCue M. Fairman A. Pramuka M. (2010). Enhancing quality of life through telerehabilitation. Phys. Med. Rehabil. Clin. N. Am. 21, 195–205. doi: 10.1016/j.pmr.2009.07.005 19951786

[ref107] Menardi A. Rossi S. Koch G. Hampel H. Vergallo A. Nitsche M. A.. (2022). Toward noninvasive brain stimulation 2.0 in Alzheimer's disease. Ageing Res. Rev. 75:101555. doi: 10.1016/j.arr.2021.101555, PMID: 34973457 PMC8858588

[ref108] Menengi̇ç K. N. Yeldan İ. Çınar N. Şahiner T. (2022). Effectiveness of motor-cognitive dual-task exercise via telerehabilitation in Alzheimer’s disease: an online pilot randomized controlled study. Clin. Neurol. Neurosurg. 223:107501. doi: 10.1016/j.clineuro.2022.107501, PMID: 36368169

[ref109] Miceli G. Laudanna A. Burani C. Capasso R. (1994). Batteria per l'Analisi dei Deficit Afasici (BADA). CEPSAG: Universita Cattolica del Sacro Cuore, Rome.

[ref110] Morris J. C. (1997). Clinical dementia rating: a reliable and valid diagnostic and staging measure for dementia of the Alzheimer type. Int. Psychogeriatr. 9, 173–176. doi: 10.1017/s1041610297004870 9447441

[ref111] Mosca I. E. Salvadori E. Gerli F. Fabbri L. Pancani S. Lucidi G.. (2020). Analysis of feasibility, adherence, and appreciation of a newly developed tele-rehabilitation program for people with MCI and VCI. Front. Neurol. 11:583368. doi: 10.3389/fneur.2020.583368, PMID: 33329326 PMC7728852

[ref112] Moyle W. (2019). The promise of technology in the future of dementia care. Nat. Rev. Neurol. 15, 353–359. doi: 10.1038/s41582-019-0188-y 31073242

[ref113] Murugaraja V. Shivakumar V. Sivakumar P. T. Sinha P. Venkatasubramanian G. (2017). Clinical utility and tolerability of transcranial direct current stimulation in mild cognitive impairment. Asian J. Psychiatr. 30, 135–140. doi: 10.1016/j.ajp.2017.09.001, PMID: 28934620

[ref114] Nissim N. R. Moberg P. J. Hamilton R. H. (2020). Efficacy of noninvasive brain stimulation (tDCS or TMS) paired with language therapy in the treatment of primary progressive aphasia: an exploratory Meta-analysis. Brain Sci. 10:597. doi: 10.3390/brainsci10090597, PMID: 32872344 PMC7563447

[ref115] Nousia A. Martzoukou M. Siokas V. Aretouli E. Aloizou A. M. Folia V.. (2021). Beneficial effect of computer-based multidomain cognitive training in patients with mild cognitive impairment. Appl. Neuropsychol. Adult 28, 717–726. doi: 10.1080/23279095.2019.1692842, PMID: 31885287

[ref116] Novelli G. Papagno C. Capitani E. Laiacona M. (1986). *Tre test clinici di ricerca e produzione lessicale Taratura su sogetti normali Archivio di psicologia, neurologia e psichiatria*.

[ref117] Nucci M. Mapelli D. Mondini S. (2012). Cognitive reserve index questionnaire (CRIq): a new instrument for measuring cognitive reserve. Aging Clin. Exp. Res. 24, 218–226. doi: 10.1007/BF03654795, PMID: 21691143

[ref118] Oldfield R. C. (1971). The assessment and analysis of handedness: the Edinburgh inventory. Neuropsychologia 9, 97–113. doi: 10.1016/0028-3932(71)90067-4 5146491

[ref119] Pagliari C. Di Tella S. Jonsdottir J. Mendozzi L. Rovaris M. De Icco R.. (2024). Effects of home-based virtual reality telerehabilitation system in people with multiple sclerosis: a randomized controlled trial. J. Telemed. Telecare 30, 344–355. doi: 10.1177/1357633x211054839, PMID: 34851211

[ref120] Palimariciuc M. Oprea D. C. Cristofor A. C. Florea T. Dobrin R. P. Dobrin I.. (2023). The effects of transcranial direct current stimulation in patients with mild cognitive impairment. Neurol. Int. 15, 1423–1442. doi: 10.3390/neurolint15040092, PMID: 38132971 PMC10745513

[ref121] Parsons T. D. (2016). Neuropsychological rehabilitation 3.0: state of the science. Clin. Neuropsychol. Technol., 113–132. doi: 10.1007/978-3-319-31075-6_7

[ref122] Pasquier F. Grymonprez L. Lebert F. Van der Linden M. (2001). Memory impairment differs in frontotemporal dementia and Alzheimer's disease. Neurocase 7, 161–171. doi: 10.1093/neucas/7.2.161, PMID: 11320163

[ref123] Peres S. C. Pham T. Phillips R. (2013). *Validation of the system usability scale (SUS) SUS in the wild*. In: Proceedings of the Human Factors and Ergonomics Society Annual Meeting: SAGE Publications Sage CA: Los Angeles, CA, pp. 192–196.

[ref124] Peretti A. Amenta F. Tayebati S. K. Nittari G. Mahdi S. S. (2017). Telerehabilitation: review of the state-of-the-art and areas of application. JMIR Rehabil. Assist. Technol. 4:e7. doi: 10.2196/rehab.7511, PMID: 28733271 PMC5544892

[ref125] Pergher V. Au J. Alizadeh Shalchy M. Santarnecchi E. Seitz A. Jaeggi S. M.. (2022). The benefits of simultaneous tDCS and working memory training on transfer outcomes: a systematic review and meta-analysis. Brain Stimul. 15, 1541–1551. doi: 10.1016/j.brs.2022.11.008, PMID: 36460294

[ref126] Petersen R. C. (2004). Mild cognitive impairment as a diagnostic entity. J. Intern. Med. 256, 183–194. doi: 10.1111/j.1365-2796.2004.01388.x 15324362

[ref127] Petersen R. C. (2011). Clinical practice. Mild cognitive impairment. N. Engl. J. Med. 364, 2227–2234. doi: 10.1056/NEJMcp0910237 21651394

[ref128] Petersen R. C. Caracciolo B. Brayne C. Gauthier S. Jelic V. Fratiglioni L. (2014). Mild cognitive impairment: a concept in evolution. J. Intern. Med. 275, 214–228. doi: 10.1111/joim.12190 24605806 PMC3967548

[ref129] Petersen R. C. Smith G. E. Waring S. C. Ivnik R. J. Tangalos E. G. Kokmen E. (1999). Mild cognitive impairment: clinical characterization and outcome. Arch. Neurol. 56, 303–308. doi: 10.1001/archneur.56.3.303 10190820

[ref130] Pillon B. Deweer B. Michon A. Malapani C. Agid Y. Dubois B. (1994). Are explicit memory disorders of progressive supranuclear palsy related to damage to striatofrontal circuits? Comparison with Alzheimer's, Parkinson's, and Huntington's diseases. Neurology 44, 1264–1270. doi: 10.1212/wnl.44.7.1264, PMID: 8035927

[ref131] Pilloni G. Vogel-Eyny A. Lustberg M. Best P. Malik M. Walton-Masters L.. (2022). Tolerability and feasibility of at-home remotely supervised transcranial direct current stimulation (RS-tDCS): single-center evidence from 6,779 sessions. Brain Stimul. 15, 707–716. doi: 10.1016/j.brs.2022.04.014, PMID: 35470019

[ref132] Pitt R. Theodoros D. Hill A. J. Russell T. (2019). The impact of the telerehabilitation group aphasia intervention and networking programme on communication, participation, and quality of life in people with aphasia. Int. J. Speech Lang. Pathol. 21, 513–523. doi: 10.1080/17549507.2018.1488990, PMID: 30200788

[ref133] Poon P. Hui E. Dai D. Kwok T. Woo J. (2005). Cognitive intervention for community-dwelling older persons with memory problems: telemedicine versus face-to-face treatment. Int. J. Geriatr. Psychiatry 20, 285–286. doi: 10.1002/gps.1282, PMID: 15717335

[ref134] Prehn K. Flöel A. (2015). Potentials and limits to enhance cognitive functions in healthy and pathological aging by tDCS. Front. Cell. Neurosci. 9:355. doi: 10.3389/fncel.2015.00355, PMID: 26441526 PMC4568338

[ref135] R Core Team (2013). R: A language and environment for statistical computing. Vienna: R Foundation for Statistical Computing.

[ref136] Rai H. Yates L. Orrell M. (2018). Cognitive stimulation therapy for dementia. Clin. Geriatr. Med. 34, 653–665. doi: 10.1016/j.cger.2018.06.010 30336993

[ref137] Realdon O. Adorni R. Ginelli D. Micucci D. Blasi V. Bellavia D.. (2023). Embedding the patient-citizen perspective into an operational framework for the development and the introduction of new Technologies in Rehabilitation Care: the Smart&Touch-ID model. Healthcare (Basel) 11:604. doi: 10.3390/healthcare11111604, PMID: 37297744 PMC10253088

[ref138] Realdon O. Rossetto F. Nalin M. Baroni I. Cabinio M. Fioravanti R.. (2016). Technology-enhanced multi-domain at home continuum of care program with respect to usual care for people with cognitive impairment: the ability-TelerehABILITation study protocol for a randomized controlled trial. BMC Psychiatry 16:425. doi: 10.1186/s12888-016-1132-y, PMID: 27887597 PMC5123349

[ref139] Rogante M. Grigioni M. Cordella D. Giacomozzi C. (2010). Ten years of telerehabilitation: a literature overview of technologies and clinical applications. NeuroRehabilitation 27, 287–304. doi: 10.3233/nre-2010-0612, PMID: 21160118

[ref140] Rönnlund M. Nyberg L. Bäckman L. Nilsson L. G. (2005). Stability, growth, and decline in adult life span development of declarative memory: cross-sectional and longitudinal data from a population-based study. Psychol. Aging 20, 3–18. doi: 10.1037/0882-7974.20.1.3, PMID: 15769210

[ref141] Rosen M. J. (2004). Telerehabilitation. Telemed. J. E Health 10, 115–117. doi: 10.1089/tmj.2004.10.115 15319039

[ref142] Rossetto F. Isernia S. Realdon O. Borgnis F. Blasi V. Pagliari C.. (2023). A digital health home intervention for people within the Alzheimer's disease continuum: results from the ability-TelerehABILITation pilot randomized controlled trial. Ann. Med. 55, 1080–1091. doi: 10.1080/07853890.2023.2185672, PMID: 36929703 PMC10030155

[ref143] Rossi S. Cappa S. F. Babiloni C. Pasqualetti P. Miniussi C. Carducci F.. (2001). Prefrontal [correction of Prefontal] cortex in long-term memory: an “interference” approach using magnetic stimulation. Nat. Neurosci. 4, 948–952. doi: 10.1038/nn0901-948, PMID: 11528428

[ref144] Rossi S. Miniussi C. Pasqualetti P. Babiloni C. Rossini P. M. Cappa S. F. (2004). Age-related functional changes of prefrontal cortex in long-term memory: a repetitive transcranial magnetic stimulation study. J. Neurosci. 24, 7939–7944. doi: 10.1523/JNEUROSCI.0703-04.2004, PMID: 15356207 PMC6729939

[ref145] Rossi S. Pasqualetti P. Zito G. Vecchio F. Cappa S. F. Miniussi C.. (2006). Prefrontal and parietal cortex in human episodic memory: an interference study by repetitive transcranial magnetic stimulation. Eur. J. Neurosci. 23, 793–800. doi: 10.1111/j.1460-9568.2006.04600.x, PMID: 16487159

[ref146] Salthouse T. A. (2011). Neuroanatomical substrates of age-related cognitive decline. J. Psychol. Bull. 137, 753–784. doi: 10.1037/a0023262, PMID: 21463028 PMC3132227

[ref147] Sandrini M. Brambilla M. Manenti R. Rosini S. Cohen L. G. Cotelli M. (2014). Noninvasive stimulation of prefrontal cortex strengthens existing episodic memories and reduces forgetting in the elderly. Front. Aging Neurosci. 6:289. doi: 10.3389/fnagi.2014.00289, PMID: 25368577 PMC4202785

[ref148] Sandrini M. Cappa S. F. Rossi S. Rossini P. M. Miniussi C. (2003). The role of prefrontal cortex in verbal episodic memory: rTMS evidence. J. Cogn. Neurosci. 15, 855–861. doi: 10.1162/089892903322370771, PMID: 14511538

[ref149] Sandrini M. Censor N. Mishoe J. Cohen L. G. (2013). Causal role of prefrontal cortex in strengthening of episodic memories through reconsolidation. Curr. Biol. 23, 2181–2184. doi: 10.1016/j.cub.2013.08.045, PMID: 24206845 PMC3824257

[ref150] Sandrini M. Cohen L. G. (2014). “Effects of brain stimulation on declarative and procedural memories” in The stimulated brain: Cognitive enhancement using non-invasive brain stimulation. ed. Kadosh R. C. (Amsterdam: Elsevier).

[ref151] Sandrini M. Manenti R. Brambilla M. Cobelli C. Cohen L. G. Cotelli M. (2016). Older adults get episodic memory boosting from noninvasive stimulation of prefrontal cortex during learning. Neurobiol. Aging 39, 210–216. doi: 10.1016/j.neurobiolaging.2015.12.010, PMID: 26923418 PMC5108058

[ref152] Sandrini M. Manenti R. Gobbi E. Rusich D. Bartl G. Cotelli M. (2019). Transcranial direct current stimulation applied after encoding facilitates episodic memory consolidation in older adults. Neurobiol. Learn. Mem. 163:107037. doi: 10.1016/j.nlm.2019.107037, PMID: 31202902

[ref153] Sandrini M. Manenti R. Sahin H. Cotelli M. (2020). Effects of transcranial electrical stimulation on episodic memory in physiological and pathological ageing. Ageing Res. Rev. 61:101065. doi: 10.1016/j.arr.2020.101065, PMID: 32275953

[ref154] Sarazin M. Berr C. De Rotrou J. Fabrigoule C. Pasquier F. Legrain S.. (2007). Amnestic syndrome of the medial temporal type identifies prodromal AD: a longitudinal study. Neurology 69, 1859–1867. doi: 10.1212/01.wnl.0000279336.36610.f7, PMID: 17984454

[ref155] Sarazin M. Chauviré V. Gerardin E. Colliot O. Kinkingnéhun S. de Souza L. C.. (2010). The amnestic syndrome of hippocampal type in Alzheimer's disease: an MRI study. J. Alzheimers Dis. 22, 285–294. doi: 10.3233/jad-2010-091150, PMID: 20847406

[ref156] Saxena V. Pal A. (2021). Role of transcranial direct current stimulation in the Management of Alzheimer's disease: a Meta-analysis of effects, adherence and adverse effects. Clin. Psychopharmacol. Neurosci. 19, 589–599. doi: 10.9758/cpn.2021.19.4.589, PMID: 34690114 PMC8553534

[ref157] Stuss D. T. Winocur G. Robertson I. H. (2008). Cognitive neurorehabilitation: Evidence and application. 2nd Edn. New York: Cambridge University Press.

[ref158] Sunderland A. Watts K. Baddeley A. D. Harris J. E. (1986). Subjective memory assessment and test performance in elderly adults. J. Gerontol. 41, 376–384. doi: 10.1093/geronj/41.3.376 3700988

[ref159] Taub E. Uswatte G. Elbert T. (2002). New treatments in neurorehabilitation founded on basic research. Nat. Rev. Neurosci. 3, 228–236. doi: 10.1038/nrn754, PMID: 11994754

[ref160] Torpil B. Pekçetin E. Pekçetin S. (2023). The effectiveness of cognitive rehabilitation intervention with the telerehabilitation method for amnestic mild cognitive impairment: a feasibility randomized controlled trial. J. Telemed. Telecare 1:541. doi: 10.1177/1357633x231189541, PMID: 37537894

[ref161] Tulving E. (1983). Elements of episodic memory. New York: Oxford University Press.

[ref162] Vaqué-Alcázar L. Mulet-Pons L. Abellaneda-Pérez K. Solé-Padullés C. Cabello-Toscano M. Macià D.. (2021). tDCS-induced memory reconsolidation effects and its associations with structural and functional MRI substrates in subjective cognitive decline. Front. Aging Neurosci. 13:695232. doi: 10.3389/fnagi.2021.695232, PMID: 34381353 PMC8350070

[ref163] Vermeij A. Claassen J. A. Dautzenberg P. L. Kessels R. P. (2016). Transfer and maintenance effects of online working-memory training in normal ageing and mild cognitive impairment. Neuropsychol. Rehabil. 26, 783–809. doi: 10.1080/09602011.2015.1048694, PMID: 26010573

[ref164] Vestergren P. Nilsson L. G. (2011). Perceived causes of everyday memory problems in a population-based sample aged 39–99. Appl. Cogn. Psychol. 25, 641–646. doi: 10.1002/acp.1734

[ref165] Woods R. T. Britton P. G. (1977). Psychological approaches to the treatment of the elderly. Age Ageing 6, 104–112. doi: 10.1093/ageing/6.2.104, PMID: 329661

[ref166] Yao S. Liu Y. Zheng X. Zhang Y. Cui S. Tang C.. (2020). Do nonpharmacological interventions prevent cognitive decline? A systematic review and meta-analysis. Transl. Psychiatry 10:19. doi: 10.1038/s41398-020-0690-4, PMID: 32066716 PMC7026127

[ref167] Yesavage J. A. Brink T. L. Rose T. L. Lum O. Huang V. Adey M.. (1982). Development and validation of a geriatric depression screening scale: a preliminary report. J. Psychiatr. Res. 17, 37–49. doi: 10.1016/0022-3956(82)90033-4, PMID: 7183759

[ref168] Yun K. Song I. U. Chung Y. A. (2016). Changes in cerebral glucose metabolism after 3 weeks of noninvasive electrical stimulation of mild cognitive impairment patients. Alzheimers Res. Ther. 8:49. doi: 10.1186/s13195-016-0218-6, PMID: 27903289 PMC5131431

[ref169] Zimerman M. Nitsch M. Giraux P. Gerloff C. Cohen L. G. Hummel F. C. (2013). Neuroenhancement of the aging brain: restoring skill acquisition in old subjects. Ann. Neurol. 73, 10–15. doi: 10.1002/ana.23761, PMID: 23225625 PMC4880032

